# Resistance to ‘*Candidatus* Liberibacter asiaticus,’ the Huanglongbing Associated Bacterium, in Sexually and/or Graft-Compatible *Citrus* Relatives

**DOI:** 10.3389/fpls.2020.617664

**Published:** 2021-01-08

**Authors:** Mônica N. Alves, Silvio A. Lopes, Laudecir L. Raiol-Junior, Nelson A. Wulff, Eduardo A. Girardi, Patrick Ollitrault, Leandro Peña

**Affiliations:** ^1^Fundo de Defesa da Citricultura, Araraquara, Brazil; ^2^Empresa Brasileira de Pesquisa Agropecuária, Cruz das Almas, Brazil; ^3^Centre de Coopération Internationale en Recherche Agronomique pour le Développement, BIOS Department, UPR amélioration génétique des espèces à multiplication végétative, Montpellier, France; ^4^Instituto de Biologia Molecular y Celular de Plantas – Consejo Superior de Investigaciones Científicas, Universidad Politécnica de Valencia, Valencia, Spain

**Keywords:** HLB, Greening, Rutaceae, *Citrus* breeding, Aurantioideae, *Microcitrus*, *Eremocitrus*

## Abstract

Huanglongbing (HLB) is the most destructive, yet incurable disease of citrus. Finding sources of genetic resistance to HLB-associated ‘*Candidatus* Liberibacter asiaticus’ (Las) becomes strategic to warrant crop sustainability, but no resistant *Citrus* genotypes exist. Some *Citrus* relatives of the family Rutaceae, subfamily Aurantioideae, were described as full-resistant to Las, but they are phylogenetically far, thus incompatible with *Citrus*. Partial resistance was indicated for certain cross-compatible types. Moreover, other genotypes from subtribe Citrinae, sexually incompatible but graft-compatible with *Citrus*, may provide new rootstocks able to restrict bacterial titer in the canopy. Use of seedlings from monoembryonic species and inconsistencies in previous reports likely due to Las recalcitrance encouraged us to evaluate more accurately these *Citrus* relatives. We tested for Las resistance a diverse collection of graft-compatible Citrinae species using an aggressive and consistent challenge-inoculation and evaluation procedure. Most Citrinae species examined were either susceptible or partially resistant to Las. However, *Eremocitrus glauca* and Papua/New Guinea *Microcitrus* species as well as their hybrids and those with *Citrus* arose here for the first time as full-resistant, opening the way for using these underutilized genotypes as Las resistance sources in breeding programs or attempting using them directly as possible new Las-resistant *Citrus* rootstocks or interstocks.

## Introduction

Huanglongbing (HLB) is the most destructive disease of citrus worldwide. Its occurrence is associated with the infection of trees with one of the following Gram-negative intracellular α-proteobacteria, ‘*Candidatus* Liberibacter asiaticus’ (Las), ‘*Ca.* L. americanus’ (Lam) or ‘*Ca.* L. africanus’ (Laf), which colonize the phloem of their host plants (Bové, 2006). Las and Lam are naturally transmitted by the Asian citrus psyllid *Diaphorina citri* Kuwayama (Sternorrhyncha: Liviidae) (Capoor et al., 1967; Yamamoto et al., 2006), while Laf is transmitted by the African citrus psyllid *Trioza erytreae* Del Guercio (Sternorrhyncha: Triozidae) (McClean and Oberholzer, 1965). In Brazil, Las and Lam have been detected (Coletta-Filho et al., 2004; Teixeira et al., 2005), Las being the most common HLB-associated bacterium, currently present in over 99.9% of all ‘Liberibacter’-positive field samples analyzed at Fundecitrus (Bassanezi et al., 2020). Las tolerates higher temperatures, reaches higher titers in *Citrus* and is more efficiently transmitted than Lam (Lopes et al., 2009, 2013), all this making the former associated with the most severe damages caused by HLB in Asia and the Americas (Gottwald et al., 2007). HLB-infected, severely affected trees produce small and irregularly shaped fruits with a thick peel that remains green. Leaves shows a blotchy mottle, yellowing and may become thicker and with enlarged veins. The canopy show a premature defoliation and dieback of twigs (Bové, 2006). In areas where no control of the insect vector is done, the severity of symptoms and disease progression in the orchards increase rapidly (Gottwald et al., 1989, 1991).

The control of HLB is based on planting of healthy trees from insect-proof nurseries, monitoring of *D. citri* and tree flushing, application of insecticides to reduce the population of the insect vector when reaching an unacceptable threshold and rapid eradication of symptomatic trees. In Brazil, since the disease was first reported in 2004, these practices have been adopted in the main citrus producing region, comprising the São Paulo State and Southwest of Minas Gerais State (Bassanezi et al., 2020). However, the cost of implementing these strategies is high and in spite of them, the incidence of the disease remains around 20% annually in the region (Fundecitrus, 2020). These palliative and preventive measures are employed for HLB management because there is neither cure nor resistant cultivars within *Citrus*, which may be used to control the disease durably.

It has been reported that some *Citrus* relatives belonging to the family Rutaceae, subfamily Aurantioideae may be partially, transiently or totally resistant to Las. In the case of some *Poncirus trifoliata* (L.) Raf. accessions the bacterium reaches inconsistent infections with low titers and uneven distribution (Folimonova et al., 2009). For *Murraya paniculata* (L.) Jack or *Swinglea glutinosa* (Blanco) Merr. (Cifuentes-Arenas et al., 2019) infection is just transient and after a few months plants become Las-free. In *Clausena excavata* Burm. F., *Glycosmis pentaphylla* (Retz.) Corr. and other Aurantioideae *Citrus* relatives the bacterium is undetectable and thus unable to replicate in the genotype under test (Ramadugu et al., 2016).

Consistent resistance to Las characterized by lack of detectable bacterial replication in germplasm sexually compatible with *Citrus* is of major interest because it may be used in sexual breeding programs aimed to generate hybrid and backcross citrus-like populations, which could provide new rootstocks or scion varieties resistant to HLB. Moreover, segregating progenies could be useful to identify genetic loci involved in the resistance trait. However, claims for resistance to Las in close relatives to *Citrus* are discrepant depending on the use of different genetic backgrounds, challenge inoculation systems, environmental conditions, plant ages, number of replicates, field vs. greenhouse tests, seedlings vs. mature plants and grafted vs. rooted stocks (Hung et al., 2000; Folimonova et al., 2009; Shokrollah et al., 2009; Feng et al., 2015; Ramadugu et al., 2016; Miles et al., 2017). For example, *Poncirus trifoliata* has been reported as resistant (Albrecht and Bowman, 2012), partially resistant (Folimonova et al., 2009) or showing different levels of resistance, recovery or delayed infection depending on the accession (Ramadugu et al., 2016). The monoembryonic, Australian native *Eremocitrus glauca* (Lindl.) Swingle as well as *Microcitrus australasica* (F. Muell.) Swingle and other *Microcitrus* species have been considered partially resistant, with transient replication or variable responses among seedlings, respectively (Ramadugu et al., 2016), representing the first results opening the way for their use in breeding programs for Las resistance, as they are cross-compatible with *Citrus* (Swingle and Reece, 1967). However, the use of seedlings for Las challenge inoculation in monoembryonic species implies that segregating individuals were actually evaluated, likely explaining their variable responses to Las (Ramadugu et al., 2016). Moreover, Las infection through *D. citri* in the field, being the natural challenge inoculation system, does not allow distinguishing resistance to the bacterium from resistance to the psyllid vector or to both vector and bacterium. Furthermore, the influence of other abiotic and biotic factors present in the field may affect seedling performance even in absence of clear Las infection (Miles et al., 2017).

Resistant *Citrus* relatives graft-compatible with *Citrus* may provide interstocks or new rootstocks (other than the widely used *P. trifoliata* accessions and their hybrids with sweet orange and grapefruit) able to restrict bacterial titer and distribution in the scion and thus potentially reduce disease damages. *Eremocitrus glauca* and several *Microcitrus* species as *M. australasica* and the ‘Sydney’ hybrid have performed well as interstocks. Moreover, *E. glauca*, *Microcitrus* types and their hybrids warrant consideration as citrus rootstocks (Bitters et al., 1964, 1977). Additionally, the most promising Aurantioideae genera for use as citrus rootstocks were *Atalantia* (some of its species previously classified in the *Severinia* genus), *Naringi*, *Citropsis*, *Limonia* (previously known as *Feronia*) and *Swinglea* (Bitters et al., 1964, 1969, 1977). Responses to Las infection have been tested for several species from these genera (Koizumi et al., 1996; Hung et al., 2000; Feng et al., 2015; Ramadugu et al., 2016; Cifuentes-Arenas et al., 2019). However, the use of seedlings from monoembryonic species (*Citropsis*), inconsistencies in results of different reports (*Limonia*), and low number of plant replications in some cases (*Naringi* and *Atalantia)*, make it advisable to perform a more accurate evaluation of these *Citrus* relatives for resistance to Las. In addition to the identification of promising germplasm to be used as parents in sexual breeding programs or as rootstocks/interstocks, a better knowledge of the distribution of the response to Las in the gene pool within Citrinae considering phylogenetic relations, would provide valuable orientation to decipher the determinants of resistance/susceptibility.

To gain more insight into Las resistance irrespective of the insect vector within close *Citrus* relatives, we have selected cross- and/or graft-compatible Aurantioideae species of the Citrinae subtribe as well as intergeneric hybrids and inoculated them with Las. A challenge and evaluation system allowing unequivocal demonstration of either resistance or susceptibility was used. For this, we have selected mature buds from each genotype of interest, and grafted them onto ‘Rangpur’ lime (*Citrus* × *limonia* Osbeck), a well-known, Las-susceptible rootstock. Challenge inoculation was performed by grafting Las-infected budwood pieces both onto the rootstock and onto the scion, so infection may come to the scion under test directly from the infected grafted budwood or from the bacterial flow moving up from the susceptible ‘Rangpur’ lime rootstock. Evaluation for Las multiplication was done regularly by qPCR over 24 months after inoculation (MAI). By using such method, we identify here which Citrinae species are the most indicated to be used as sources of full-resistance to Las either in breeding programs or directly as potential *Citrus* rootstocks/interstocks. The distribution of susceptibility/resistance responses to Las within the Citrinae germplasm is discussed according to chloroplast-based phylogeny.

## Materials and Methods

### Plant Genotypes

**FIGURE 1:**
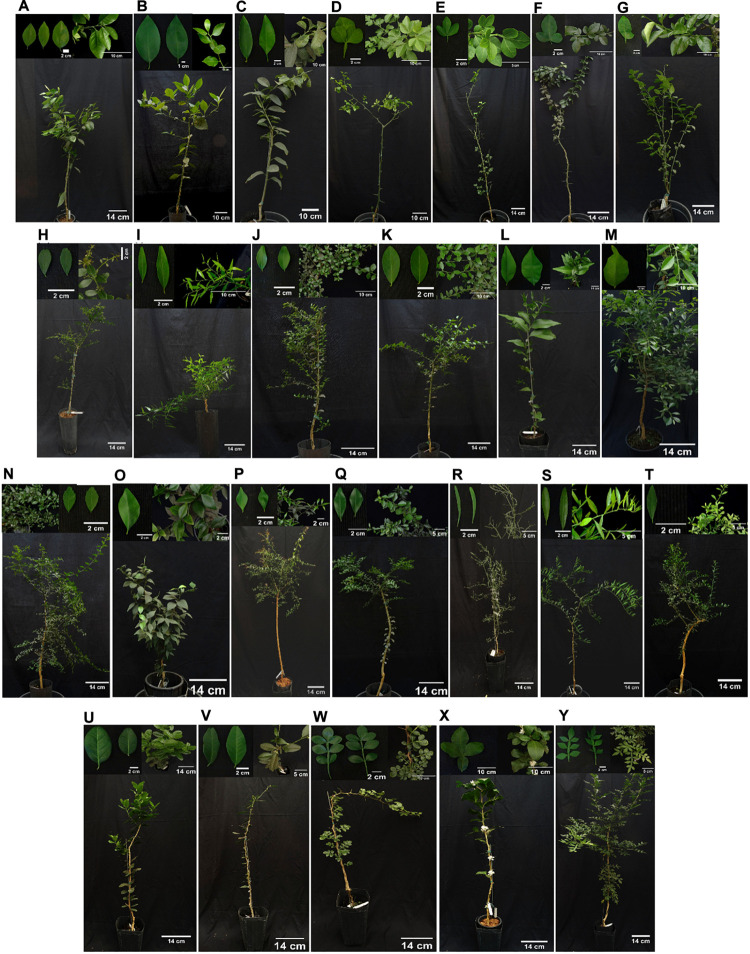
Representative photographs of each of the selected Citrinae genotypes/accessions [cross-compatible **(A–T)**; and cross-incompatible, putative graft-compatible **(U–Y)** with *Citrus* about 1 year after grafting onto ‘Rangpur’ lime rootstock]. **(A)** *Citrus* × *sinensis* ‘Pera’; **(B)** *C.* × *sinensis* ‘Tobias’; **(C)** *C. halimii;* **(D)** *Poncirus trifoliata* ‘Pomeroy’; **(E)** *P. trifoliata* ‘Benecke’; **(F)** *P. trifoliata* ‘Barnes’; **(G)** *P. trifoliata* ‘Rubidoux’; **(H)** *Microcitrus australasica;* **(I)** *M. australasica* ‘Sanguinea’; **(J)** *M. australasica* ‘True Sanguinea’; **(K)** ‘Faustrimedin’ hybrid; **(L)** *M. inodora;* **(M)** *M. warburgiana;* **(N)** *M. papuana;* **(O)** *M. australis;* **(P)** *M. virgata* hybrid; **(Q)** *Microcitrus* sp. × *E. glauca* hybrid; **(R)** *Eremocitrus glauca;* **(S)** *E. glauca* × *C.* × *sinensis* hybrid; **(T)** *E. glauca* × *Microcitrus* sp. hybrid; **(U)** *Atalantia citroides*; **(V)** *A. ceylanica*; **(W)** *Limonia acidissima*; **(X)** *Citropsis gilletiana*; **(Y)** *Naringi crenulata*.

The cross-compatible *Citrus* relatives selected were four *Poncirus trifoliata* (L.) Raf. accessions, ‘Pomeroy,’ ‘Rubidoux,’ ‘Barnes,’ and ‘Benecke,’ commonly used as rootstocks and in breeding programs with *Citrus* to generate new hybrid rootstocks (Phillips and Castle, 1977; Soares Filho et al., 2003; Bowman et al., 2016) and belonging to two different genetic groups, ‘Rubidoux’ and ‘Barnes’ to group 3 and ‘Pomeroy’ and ‘Benecke’ to group 4, according to Fang et al. (1997); the Australian limes, including (1) the pure species: *Microcitrus australasica* (F. Muell.) Swingle, *M. australasica* ‘Sanguinea,’ *M. australasica* ‘True Sanguinea,’ *M. inodora* (F.M. Bail) Swingle, *M. warburgiana* (F.M. Bailey) Tanaka, *M. papuana* Winters, *M. australis* (A. Cunn. ex Mudie) Swingle and *Eremocitrus glauca* (Lindl.) Swingle, (2) three hybrids among them: *M. virgata* (*M. australis* × *M. australasica*), known as the ‘Sydney’ hybrid, a *Microcitrus* sp. × *E. glauca* and an *E. glauca* × *Microcitrus* sp. hybrids, and (3) two Australian lime hybrids with *Citrus*: an *Eremocitrus glauca* × *Citrus* × *sinensis* hybrid (eremorange) and the ‘Faustrimedin’ hybrid [*M. australasica* × ‘Calamondin’ (*Fortunella* sp. × *C. reticulata* Blanco); *C.* × *oliveri*] (Table 1 and Figure 1). Among those Citrinae genotypes cross-incompatible but graft-compatible with *Citrus*, we selected those recommended as suitable rootstocks and/or interstocks by Bitters et al. (1964, 1969, 1977), including *Atalantia citroides* Pierre ex Guillaumin, *A. ceylanica* (Arn.) Oliv., *Limonia acidissima* L., *Citropsis gilletiana* Swingle & M. Kellerm, and *Naringi crenulata* (Roxb.) Nicolson. *Limonia acidissima* is classified in the Balsamocitrinae subtribe by Swingle and Reece (1967). However, it appears to be closely related with *Atalantia* species according to chloroplastic phylogenetic studies and clearly included in the Citrinae clade (Bayer et al., 2009). As Las susceptible controls, we used two *Citrus* genotypes, the Brazilian sweet orange varieties ‘Pera’ and ‘Tobias’ [*C.* × *sinensis* (L.) Osbeck] (Donadio et al., 1995). We also added the ‘Mountain’ citron (*C. halimii* B.C. Stone), which was the less Las-susceptible type within *Citrus* according to Ramadugu et al. (2016) (Table 1 and Figure 1).

**TABLE 1:** Citrinae genotypes/accessions tested for resistance to ‘*Candidatus* Liberibacter asiaticus.’

Genotype/Accession^a^	Common name^a^
*Citrus* × *sinensis* (L.) Osbeck ‘Pera’ **(P^c^)**	‘Pera’ sweet orange
*C.* × *sinensis* ‘Tobias’ **(P)**	‘Tobias’ sweet orange
*Citrus halimii* B.C. Stone **(M^d^)**	‘Mountain’ citron
*Poncirus trifoliata* (L.) Raf. ‘Pomeroy’ **(P)**	‘Pomeroy’ trifoliate orange
*P. trifoliata* ‘Benecke’ **(P)**	‘Benecke’ trifoliate orange
*P. trifoliata* ‘Barnes’ **(P)**	‘Barnes’ trifoliate orange
*P. trifoliata* ‘Rubidoux’ **(P)**	‘Rubidoux’ trifoliate orange
*Microcitrus australasica* (F. Muell.) Swingle **(M)**	Australian finger lime
*M. australasica* ‘Sanguinea’ **(M)**	‘Sanguinea’ Australian finger lime
*M. australasica* ‘True Sanguinea’ **(M)**	‘True Sanguinea’ Australian finger lime
*M. australasica* × (*Fortunella* sp. × *Citrus reticulata*) ‘Calamondin’; *C.* × *oliveri* **(PM^e^)**	‘Faustrimedin’ hybrid
*M. inodora* (F.M. Bail) Swingle **(M)**	Australian large-leaf wild lime
*M. warburgiana* (F.M. Bailey) Tanaka **(M)**	New Guinean wild lime
*M. papuana* Winters **(M)**	Brown river finger lime
*M. australis* (A. Cunn. Ex Mudie) Swingle **(M)**	Australian round lime
*M. virgata (M. australis* × *M. australasica)* **(PM)**	‘Sydney’ hybrid
*Microcitrus* sp. × *E. glauca* hybrid **(PM)**	Australian lime hybrid BGC 695^b^
*E. glauca* (Lindl.) Swingle **(M)**	Australian desert lime
*E. glauca* × *C.* × *sinensis* hybrid **(PP)**	Eremorange
*E. glauca* × *Microcitrus* sp. hybrid **(PM)**	Australian desert lime hybrid BGC 682^b^
*Atalantia citroides* Pierre ex Guillaumin **(PP^f^)**	Cochin China atalantia
*A. ceylanica* (Arn.) Oliv. **(PP)**	Ceylon atalantia
*Limonia acidissima* L. **(P)**	Indian wood apple or elephant apple
*Citropsis gilletiana* Swingle & M. Kellerm. **(M)**	Gillet’s cherry orange
*Naringi crenulata* (Roxb.) Nicolson **(PM)**	Hesperethusa

*^a^The nomenclature used follows Swingle and Reece (1967) and Bayer et al. (2009).*

*^b^Original accession number at the Citrus Germplasm Bank (BGC) of Embrapa Cassava & Fruits in Cruz das Almas, Bahia.*

*^c^P, polyembryonic; ^d^M, monoembryonic; ^e^PM, possibly monoembryonic; ^f^PP, possibly polyembryonic; according to Swingle and Reece (1967) and Bitters (1986).*

### Plant Material, Grafting and Las Challenge Inoculation

In a preliminary experiment, the 25 accessions from *Citrus* and *Citrus* relatives shown in Table 1, belonging to the family Rutaceae, subfamily Aurantioideae, subtribe Citrinae, were selected from the virus/viroid-free Fundecitrus germplasm collection and propagated by grafting onto ‘Rangpur’ lime (*Citrus* × *limonia* Osbeck) nucellar rootstocks to study graft-compatibility. The Las-susceptible, graft-compatible sweet orange varieties ‘Pera’ and ‘Tobias’ [*C.* × *sinensis* (L.) Osbeck] (Donadio et al., 1995) were also propagated on ‘Rangpur’ lime as controls.

Twenty to thirty plants per accession from Citrinae genotypes that were graft-compatible with ‘Rangpur’ lime were propagated on this same rootstock using buds from a single donor mother plant per genotype, and kept at a greenhouse in Araraquara, São Paulo State, Brazil (Figure 1). True-to-typeness was assessed by analyzing all Citrinae species morphologically (Swingle and Reece, 1967) and in the case of Australian lime hybrids also by using SSR molecular markers (Fanciullino et al., 2005; Froelicher et al., 2008; Luro et al., 2008; Ollitrault et al., 2008) (Supplementary Table 1). Plants were grown in 4 L polyethylene bags filled with coir, irrigated and fertilized twice a week, and sprayed monthly with insecticides and miticides. Las challenge-inoculation experiments were conducted in a greenhouse in which the mean daily air temperature varied between 18.5°C to 34.4°C and illumination was natural.

The original source of inoculum was Las-positive sweet orange budwood from a farm located in São Paulo (Brazil), propagated and kept at Fundecitrus since 2006 (Lopes et al., 2009). Las-free and Las-positive *D. citri* populations are continuously reared at Fundecitrus, as described in Parra et al. (2016). Las-free psyllids are reared on healthy *Murraya paniculata* (L.) Jack seedlings while Las-positive psyllids are reared on Las-positive ‘Valencia’ sweet orange plants grafted on ‘Rangpur’ lime. For this work, greenhouse-grown ‘Rangpur’ lime seedlings were inoculated by exposing them to Las-infected *D. citri* insects. Budwood from these seedlings was then used to inoculate ‘Valencia’ sweet orange nursery plants grafted on ‘Rangpur’ lime, which were used as source of inoculum for the 25 Citrinae accessions. Las-infection in ‘Rangpur’ lime seedlings and ‘Valencia’ sweet orange plants was confirmed by qPCR (Li et al., 2006). Seven to sixteen plants per genotype were selected based on regular and homogeneous growth within each accession to be graft-inoculated with Las using two budwood pieces ca. 3 to 5 cm long per plant.

In all plants to be tested, one Las-infected (qPCR-positive) budwood piece was grafted on the ‘Rangpur’ lime rootstock, and another one on the scion variety, both at 5 cm below and above the scion-rootstock junction, respectively. At the same time, 4 to 14 uniform plants of each genotype were grafted with Las-negative budwood from healthy greenhouse-grown ‘Valencia’ sweet orange plants grafted on ‘Rangpur’ lime, which were used as negative controls. In the case of ‘Pera’ sweet orange, 41 plants were Las graft-inoculated as described above and 33 plants were grafted with healthy budwood pieces. Three months after graft challenge inoculation, plants were pruned at 0.5–1.5 m from the rootstock to promote new shoot growth and thus Las translocation from the infected budwood pieces and rootstock to the scion in the Las-challenge inoculated plants (Johnson et al., 2014; Raiol-Junior et al., 2021). Control plants were pruned likewise. Only those plants showing Las infection in the ‘Rangpur’ lime rootstock at 12 months after graft-inoculation (MAI) were considered as successfully Las-inoculated. The exact number of plants used per genotype for resistance evaluation as well as that of healthy controls are detailed in Supplementary Table 2.

### Sample Collection and Evaluation by qPCR

A random but representative sampling of 16–20 leaf pieces from actively growing shoots per plant scion were collected at 4, 6, 8, 10, and 12 MAI with Las-infected or Las-free (control) budwood. Those accessions that remained with few or no Las-positive scions at 12 MAI, and continued to grow well, were re-evaluated at 24 MAI, including *Microcitrus australasica* ‘True Sanguinea,’ *M. warburgiana*, *M. papuana*, *M. australis*, *Microcitrus* sp. × *E. glauca* hybrid, *E. glauca* × *C.* × *sinensis* hybrid and *E. glauca* × *Microcitrus* sp. hybrid as well as *C.* × *sinensis* ‘Tobias’ and ‘Pera’ controls.

To be sure that Las challenge-inoculated plants were actually infected, the fibrous root tissue from the susceptible ‘Rangpur’ lime rootstock was evaluated at 12 MAI as well as its bark at 12 and 24 MAI. Only plants with Las-positive roots at 12 MAI had their leaves evaluated. Las graft-infection success was calculated as the percentage of plants with Las-positive rootstocks per the total number of Las-graft-inoculated composite plants (Supplementary Table 2).

To assess movement of Las from the rootstock to the scion through the vascular system, bark samples were collected at 5 and 30 cm above the bud union and at the scion canopy (21–152 cm) from each plant that remained without Las-multiplication in leaf samples including *M. warburgiana*, *M. papuana*, *M. australis*, *Microcitrus* sp. × *E. glauca* hybrid, *Eremocitrus glauca*, *E. glauca* × *C.* × *sinensis* hybrid and *E. glauca* × *Microcitrus* sp. hybrid as well as *C.* × *sinensis* ‘Tobias’ and ‘Pera’ as Las positive controls.

Samples were all subjected to DNA extraction. Total DNA was extracted from 0.5 g of leaf midribs, 0.3 g of fibrous roots or 0.3 g of bark tissue and processed using a cetyltrimethylammonium bromide (CTAB) extraction buffer (Murray and Thompson, 1980) as described by Teixeira et al. (2005). DNA quality was checked with the NanoDrop (Thermo Scientific) (Desjardins and Conklin, 2010). Real-time polymerase chain reactions (qPCR) for detection of the 16S rDNA from Las were ran using 1 μL of total DNA (100 ng/μL), TaqMan^®^ PCR Master Mix (1x) (Invitrogen, Carlsbad, CA, United States) and HLB as Las-specific primer/probe (0.5 μM/0.2 μM) in a StepOnePlus thermocycler (Applied Biosystems) as described by Li et al. (2006). A positive and a negative sample were included as quality controls during DNA extractions and qPCR assays. As an internal control, the mitochondrial gene cytochrome oxidase (COX) was used (Li et al., 2006). As plant housekeeping controls, primers from the Actin and Rubisco small subunit genes were designed based on homologous sequences of six and nine different plant species, respectively, that were aligned using DNA MAN^®^, and were also used (Supplementary Tables 3, 4).

Leaf, bark and root samples were considered Las-positive when their qPCR cycle threshold (Ct) was lower than 34.0. Las quantification was done based on the linear relation among Ct and the 16S rRNA log, according to *y* = −0.2998Ct + 11.042, *R*^2^ = 0.9981 (Lopes et al., 2013). Target gene concentrations below 0.9 16S rRNA log, which corresponded to a Ct value of 34.0, produced variable results (Lopes et al., 2013), so this was used as the lower detection limit for Las-positive samples. However, Ct values between 34.0 and 36.0 were considered as suspicious to be positive, so only when all samples from other remaining time points showed Ct values over 36.0, the plant was considered as Las-negative. For root samples, a standard curve was generated using the amplified PCR product of the gene 16S rRNA from Las. Eight- to ten-fold serial dilutions were prepared in triplicate and mixed in total DNA at 100 ng/μl for healthy ‘Rangpur’ lime roots (Supplementary Figure 1). The linear relation among Ct and the log 16S rRNA was *y* = 1.073Ct + 25.098, *R*^2^ = 0.9929. Ct values higher than 34.0 were also inconsistent for roots. When Ct values were 34.0 or close to 34.0, bark pieces from the rootstock were further analyzed at 12 and 24 MAI by qPCR to confirm Las-infection of the ‘Rangpur’ lime.

### Data Analysis

Data were analyzed with the statistical software RStudio (RStudio Team, 2015). The data were assessed for homoscedasticity (Levene, 1960), and normality (Shapiro and Wilk, 1965). All multiple comparisons were first subjected to analysis of variance (ANOVA). Contrast analysis was performed to compare means between groups (Rosenthal and Rosnow, 1985) and when significant differences were found, the means were compared by the *t*-test (*p* < 0.05). Analysis was done using the Las titer average at 12 MAI in log10 of amplicon copies per gram of plant tissue estimated based on a standard curve described by Lopes et al. (2013).

### Phylogenetic Tree Construction

To establish the phylogenetic tree, we used the sequences of eight chloroplastic regions (atpB-coding region, rbcL-atpB spacer, rps16 spacer, trnL-F region, rps4-trnT spacer, matK-5′trnK spacer, psbM-trnDGUC spacer and trnG intron) published by Bayer et al. (2009) selecting those accessions for which informations on HLB resistance were available from our study and that from Ramadugu et al. (2016). We added a few accessions within the True Citrus group to cover more species as well as some representatives of the Triphasiinae subtribe to maintain the global structure of the Aurantioideae phylogenetic tree. According to the Swingle and Reece (1967) classification, the Clauseneae tribe was represented by three subtribes: Clauseninae (six accessions), Merrilliinae (one accession) and Micromelinae (one accession). The Citreae tribe was represented by three subtribes: Triphasilinae (nine accessions), Balsamocitrinae (five accessions) and Citrinae (37 accessions). For the 59 selected accessions (Supplementary Table 5), the sequences were obtained from the National Center of Biotechnology Information (NCBI). For each genome fragment, the sequences were aligned to the *C.* × *sinensis* reference chloroplast genome sequence (GenBank: NC008334) using BioEdit software (Hall, 1999) and curated manually in InDel areas. The resulting alignment was used to establish a Neighbor Joining (NJ) tree according to Kimura (1980) genetic distances and considering deletions as missing data, using DarWin 6 software (Perrier and Jacquemoud-Collet, 2006). One thousand bootstraps were performed to test the robustness of each branching.

## Results

### Graft-Compatibility Onto ‘Rangpur’ Lime Rootstock

**FIGURE 2:**
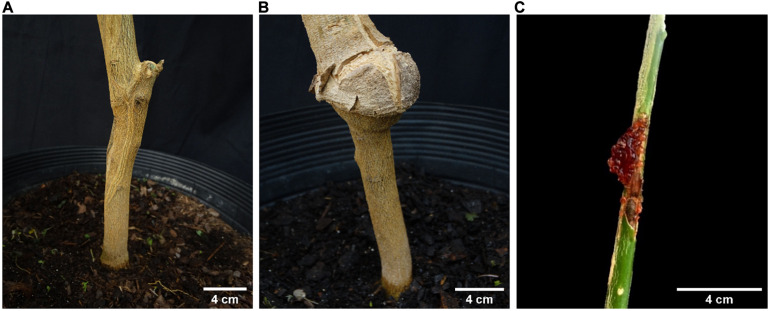
**(A)** An *Eremocitrus glauca* × *Citrus* × *sinensis* scion propagated on ‘Rangpur’ lime as an example of good rootstock/scion compatibility between two genotypes. **(B)** *Limonia acidissima* scion propagated on ‘Rangpur’ lime showing overgrowth incompatibility at the graft union. **(C)** Profuse gum exudation in the graft union between ‘Valencia’ sweet orange budwood and *Citrus halimii* scion.

Most genotypes used were graft-compatible on ‘Rangpur’ lime (Figure 2A). Incompatibility reactions were observed in two species, *L. acidissima* and *C. halimii*, but only about 1 to 2 years after propagating them and once they had been already Las-inoculated. For *L. acidissima*, progressive overgrowth of the scion resulted in five of the 10 Las challenge-inoculated plants dying before the twelfth month of evaluation (Figure 2B). Even considering that two plants were positive (Ct ≤ 34.0) and another one was considered suspicious (Ct > 34 and ≤36.0) at 10 MAI, the insufficient number of replications alive at 12 MAI, led us to disregard this species in our analysis. At the end of the experiment, all grafts of these accessions were affected by overgrowth of the scion. For *C. halimii*, incompatibility reaction took almost 1 year to start appearing, but in this case, it affected to 100% of the grafts of ‘Valencia’ sweet orange budwood pieces on the scion but not on the ‘Rangpur’ lime rootstock, neither on the bud union between *C. halimii* and ‘Rangpur’ lime. It was characterized by a profuse exudation of gum at the graft union in all plants (Figure 2C). Therefore, only after being propagated, inoculated, and analyzed by qPCR for Las infection during months, we realized that these plants had a problem which was seriously affecting their growth and general aspect and which finally killed all of them between 11 and 15 MAI (Supplementary Table 6). The ‘Rangpur’ lime rootstocks that survived resulted Las-positive at 12 MAI. Moreover, all their *L. acidissima* and *C. halimii* scions were also Las-positive at 12 MAI (results not shown; Supplementary Table 6). A possible role of Las in these incompatibility reactions could be discarded because uninfected budwood controls suffered the same incompatibility problems with identical frequencies for both genotypes (results not shown).

### Response of Graft-Compatible Citrinae Species to Challenge-Inoculation of Las

All graft-compatible accessions were propagated on ‘Rangpur’ lime rootstock and challenge-inoculated by grafting two budwood pieces, one on the rootstock and another one on the scion, from Las-infected ‘Valencia’ sweet orange trees. Uninfected, healthy ‘Valencia’ trees were used as a source of budwood pieces to be grafted in the corresponding controls for each genotype. Las infection in budwood pieces was confirmed by testing a small piece from each one by qPCR. Using this challenge inoculation system, Las infection in the scion may come from the infected budwood pieces grafted on it and/or from the ‘Rangpur’ lime stock once infected. In any case, infection of the rootstock would ensure a continuous flow of bacteria moving up to the scion. Root infection was evaluated just at 12 MAI, because root sampling damaged the composite plants and at that time-point scion Las-infection outcomes were already obtained. To confirm consistent rootstock infection, ‘Rangpur’ lime bark was also evaluated at 12 MAI. However, Las infection in the rootstock was successful in 63 to 100% of the graft-inoculated plants, irrespective of the phylogenetic relation of scion types with *Citrus* (Supplementary Table 2). As all scions grafted on challenge inoculated but Las-negative ‘Rangpur’ lime rootstocks resulted to be Las-negative, for further analyses of Las-resistance we only considered those plants with Las-positive rootstocks. This result revealed largely inefficient Las-infection of the scions through graft-inoculation of infected budwood and how difficult was to get 100% infection of a well-known Las host as ‘Rangpur’ lime even under controlled experimental conditions and forced challenge-inoculations, and therefore how important is to use proper controls of infection to avoid getting false negatives when testing genotypes for resistance to this bacterium.

Based on Las infection results, we classified the Citrinae genotypes used as scions in three categories (Table 2):

**TABLE 2:** ‘*Candidatus* Liberibacter asiaticus’ infection in the Citrinae genotypes evaluated as determined through detection of the 16S DNA by qPCR at 12 months after infection.

Category	Accession	Freq.^a^	Scion		Rootstock			
			Leaves		Root		Bark	
			Ct avg^b^ ± SEM^c^	Log avg^d^ ± SEM	Ct avg ± SEM	Log avg ± SEM	Ct avg ± SEM	Log avg ± SEM
1	*Citrus* × *sinensis* ‘Pera’	41/41	25.7 ± 0.6	4.7 ± 0.2	30.0 ± 0.6	3.4 ± 0.2	27.4 ± 0.4	4.2 ± 0.1
	*C.* × *sinensis* ‘Tobias’	09/09	22.3 ± 0.4	5.7 ± 0.1	29.9 ± 1.1	3.4 ± 0.3	29.6 ± 0.9	3.5 ± 0.3
	*Citrus* × *halimii*	05/05	30.0 ± 1.1	3.4 ± 0.3	31.4 ± 0.9	3.0 ± 0.3	30.6 ± 0.3	3.2 ± 0.1
	*Poncirus trifoliata* ‘Pomeroy’	06/06	27.9 ± 1.4	4.0 ± 0.4	30.6 ± 1.5	3.2 ± 0.5	31.0 ± 0.4	3.1 ± 0.1
	*P. trifoliata* ‘Benecke’	06/06	31.2 ± 1.0	3.0 ± 0.3	29.7 ± 1.4	3.5 ± 0.4	29.5 ± 0.8	3.5 ± 0.2
	*P. trifoliata* ‘Barnes’	08/08	32.0 ± 1.1	2.8 ± 0.3	31.0 ± 0.9	3.1 ± 0.3	29.6 ± 1.0	3.5 ± 0.3
	*P. trifoliata* ‘Rubidoux’	06/06	26.2 ± 1.4	4.5 ± 0.4	30.6 ± 1.1	3.2 ± 0.3	29.4 ± 0.9	3.6 ± 0.3
	*Atalantia citroides*	10/10	29.9 ± 0.7	3.4 ± 0.2	26.0 ± 0.9	4.6 ± 0.3	26.2 ± 0.6	4.5 ± 0.2
	*A. ceylanica*	12/12	29.4 ± 1.2	3.6 ± 0.4	25.8 ± 0.4	4.7 ± 0.1	26.0 ± 0.7	4.6 ± 0.2
2	*Microcitrus australasica*	06/11*	31.6 ± 0.7	2.9 ± 0.2	27.4 ± 0.6	4.1 ± 0.2	30.8 ± 0.5	3.2 ± 0.2
	*M. australasica* ‘Sanguinea’	04/09*	26.0 ± 0.9	4.6 ± 0.3	29.0 ± 1.4	3.7 ± 0.4	28.7 ± 0.6	3.6 ± 0.1
	*M. australasica* ‘True Sanguinea’	01/11*	33.8 ± 0.0	2.3 ± 0.0	26.8 ± 0.0	4.4 ± 0.0	29.3 ± 0.0	3.6 ± 0.0
	Faustrimedin hybrid; *C*. × *oliveri*	02/10*	33.1 ± 0.9	2.5 ± 0.3	33.5 ± 0.6	2.4 ± 0.2	30.8 ± 0.2	3.2 ± 0.1
	*Microcitrus inodora*	04/12*	25.4 ± 2.9	4.8 ± 0.9	31.6 ± 1.4	2.9 ± 0.4	28.0 ± 1.0	4.0 ± 0.3
	*Microcitrus virgata* hybrid	03/10*	30.0 ± 1.7	3.4 ± 0.5	30.1 ± 1.9	3.4 ± 0.6	31.9 ± 1.1	2.8 ± 0.4
	*Citropsis gilletiana*	05/13*	32.2 ± 1.9	2.7 ± 0.6	24.8 ± 0.3	5.0 ± 0.1	27.6 ± 0.5	4.1 ± 0.4
	*Naringi crenulata*	05/09*	32.1 ± 0.7	2.7 ± 0.2	26.8 ± 0.3	4.4 ± 0.1	28.2 ± 0.7	3.8 ± 0.2
3	*Microcitrus warburgiana*	00/09	nd^e^	nd	31.9 ± 0.6	2.8 ± 0.2	30.1 ± 0.6	3.4 ± 0.2
	*Microcitrus papuana*	00/08	nd	nd	29.1 ± 0.8	3.7 ± 0.2	30.0 ± 0.5	3.4 ± 0.1
	*Microcitrus australis*	00/10	nd	nd	32.5 ± 0.5	2.7 ± 0.2	30.7 ± 0.7	3.2 ± 0.2
	*Microcitrus* × *Eremocitrus* hybrid	00/07	nd	nd	31.8 ± 0.7	2.8 ± 0.2	29.4 ± 0.7	3.6 ± 0.2
	*Eremocitrus glauca*	00/07	nd	nd	25.3 ± 0.3	4.8 ± 0.1	29.8 ± 0.8	3.5 ± 0.2
	*E. glauca* × *C.* × *sinensis* hybrid	00/12	nd	nd	30.7 ± 0;4	3.2 ± 0.1	28.9 ± 0.5	3.7 ± 0.1
	*Eremocitrus* × *Microcitrus* hybrid	00/08	nd	nd	33.1 ± 0.3	2.5 ± 0.1	31.1 ± 0.3	3.1 ± 0.1

*^a^Freq., number of Las-positive scions (Ct ≤ 34.0)/total number of plants evaluated (with Las-positive rootstocks, Ct ≤ 34.0).*

*^b^Ct avg, cycle threshold average determined through detection of the 16S DNA by qPCR.*

*^c^SEM, standard error of the mean.*

*^d^Log, Las titer average in log10 of amplicon copies per gram of plant tissue estimated based on a standard curve as described by Lopes et al. (2013).*

^e^nd, non-detected.

*Rootstock roots and bark avg ± SEM data from accessions in the category 2 were obtained exclusively from plants with positive (Ct ≤ 34.0) or suspicious to be positive (34.0 < Ct ≤ 36.0) leaves. Details on values of each plant are in the Supplementary Tables 6, 7, 9.

#### Category 1. Susceptible

**FIGURE 3:**
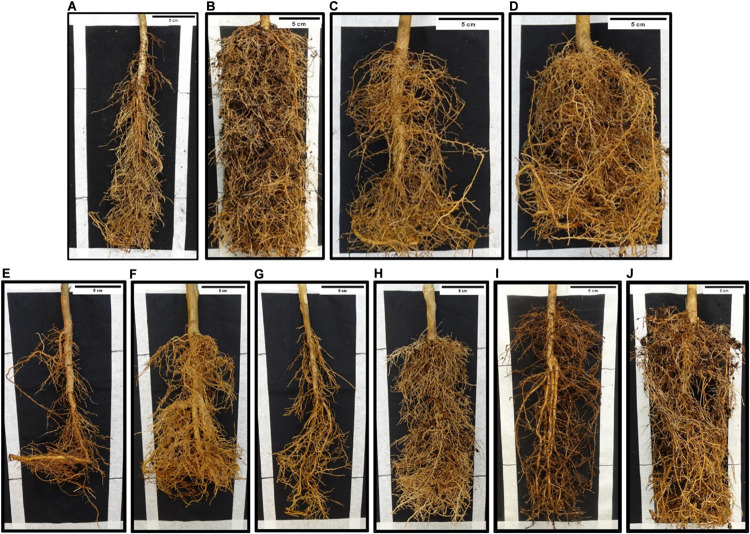
HLB-like symptoms in roots from ‘Rangpur’ lime rootstocks graft-inoculated either with Las-infected or healthy control budwood in composite plants with infected vs. healthy control scions from three susceptible, the partially resistant *Microcitrus australasica* and the full-resistant *E. glauca* × *C.* × *sinensis* genotypes at 12 months after challenge-inoculation. **(A)** *Citrus* × *sinensis* ‘Pera’, Las-infected; **(B)** *C.* × *sinensis* ‘Pera’, control; **(C)** *C.* × *sinensis* ‘Tobias’, Las-infected; **(D)** *C.* × *sinensis* ‘Tobias’, control; **(E)** *Atalantia citroides*, Las-infected*;* **(F)** *Atalantia citroides*, control*;* **(G)** *Microcitrus australasica*, Las-infected; **(H)** *Microcitrus australasica*, control; **(I)** *E. glauca* × *C.* × *sinensis* hybrid, Las-inoculated; **(J)** *E. glauca* × *C.* × *sinensis* hybrid, control.

This comprised all genotypes with 100% of the clonal propagations resulting Las-positive at 12 MAI, when their evaluation finalized. It included nine genotypes: the two sweet orange controls ‘Pera’ and ‘Tobias,’ *C. halimii*, the four accessions of *P. trifoliata* (‘Pomeroy,’ ‘Benecke,’ ‘Barnes,’ and ‘Rubidoux’), and the two *Atalantia* species (*A. citroides* and *A. ceylanica*). Ct values in the scion canopy leaves and ‘Rangpur’ lime roots and bark for each plant and evaluation date are detailed in Supplementary Table 6. HLB-like symptoms on the infected scions at 10–12 MAI were masked by nutritional deficiencies probably due to the severe root loss caused by the bacterium (Figure 3), so sensitivity to Las infection in the scion was not evaluated. Because of the severe root damage, one *A. citroides* composite plant died about 10 MAI. As mentioned above, *C. halimii* scions were seriously affected by an incompatibility problem with ‘Valencia’ budwood pieces, irrespective of being infected or not with Las, which finally killed four infected composite plants at 11–12 MAI and the remaining infected ones before 15 MAI. *C. halimii* non-inoculated control composite plants, which had been grafted with Las-free ‘Valencia’ budwood pieces, died between 11 and 14 months after grafting.

The contrast analysis showed not significant differences in bacterial titers when comparing all Category 1 accessions together (*p* < 0.0884), and *Citrus* with *P. trifoliata* accessions (*p* < 0.12). However, they were significant when comparing *P. trifoliata* versus *Atalantia* (*p* < 0.000183) and also when comparing *Citrus* versus *Atalantia* accessions (*p* < 1e−04).

#### Category 2. Partially Resistant

Eight Citrinae accessions were considered as partially resistant, because they showed Las infection in just part of the clonally propagated scions. In Las-positive propagations, infection was usually delayed and bacterial titers were generally lower than those found in sweet orange controls (*p* < 0.0377). The three accessions of Australian finger lime (*Microcitrus australasica, M. australasica* ‘Sanguinea’ and *M. australasica* ‘True Sanguinea’), ‘Faustrimedin,’ *M. inodora, M. virgata*, the cherry orange *Citropsis gilletiana* and *Naringi crenulata* were included in this group (Table 2 and Supplementary Table 7). Such variable responses may be considered as a genetic resistance trait, which was partially overcome in some clonal propagations by aggressive Las challenge inoculation. However, no correlation was found between higher bacterial titer in the rootstock and resistance breaking in the scion (Supplementary Table 8).

The three *M. australasica* accessions showed similar response patterns, with six out of 11 scions being Las-positive at 8 MAI, and the other five by 12 MAI. However, Las infection was inconsistent because some scions that were infected at 8 MAI resulted qPCR-negative at 12 MAI. Moreover, five scions resulted Las-positive, with Ct > 32.0, just at 1–2 out of five time points evaluated (Supplementary Table 7). As Las titers were generally low, with Ct > 30.0 at 10–12 MAI, inconsistent detection may be derived from uneven distribution of a few bacterium cells infecting those scions. The ‘Sanguinea’ accession showed four out of nine Las positive scions but the ‘True Sanguinea’ showed just one (Ct > 30.0) out of 11 scions (Supplementary Table 7). Because of its apparent higher resistance compared to the other accessions, we decided to maintain and re-evaluate the ‘True Sanguinea’ accession again at 24 MAI. At this time, three out of 10 were Las-positive (Ct > 32.0). The remaining one died possibly due to the severe damages caused by the bacterium to the ‘Rangpur’ lime rootstock (Table 3 and Supplementary Table 10).

**TABLE 3:** ‘*Candidatus* Liberibacter asiaticus’ infection in the Citrinae genotypes re-evaluated at 24 months after inoculation, as determined through detection of the 16S rDNA by qPCR.

Category	Accession	Freq.^a^	Scion		Rootstock	
			Leaves		Bark	
			Ct avg^b^ ± SEM^c^	Log avg^d^ ± SEM	Ct avg ± SEM	Log avg ± SEM
1	*Citrus* × *sinensis* ‘Pera’	15/15	25.5 ± 0.7	4.7 ± 0.2	27.9 ± 0.5	4.0 ± 0.1
	*C.* × *sinensis* ‘Tobias’	09/09	23.8 ± 1.4	5.2 ± 0.4	29.1 ± 0.5	3.7 ± 0.2
2	*M. australasica* ‘True Sanguinea’	03/10*	33.0 ± 0.1	2.5 ± 0.0	30.9 ± 0.7	3.1 ± 0.2
	Faustrimedin hybrid; *C*. × *oliveri*	04/10*	30.3 ± 1.1	3.3 ± 0.3	31.2 ± 1.1	3.0 ± 0.3
	*Microcitrus inodora*	03/07*	25.3 ± 1.7	4.8 ± 0.5	27.4 ± 1.3	4.2 ± 0.4
	*Microcitrus virgata* hybrid	00/09*	nd^e^	nd	29.0 ± 0.4	3.7 ± 0.1
3	*Microcitrus warburgiana*	00/06	nd	nd	30.1 ± 0.0	3.4 ± 0.0
	*Microcitrus papuana*	00/04	nd	nd	31.2 ± 0.8	3.0 ± 0.2
	*Microcitrus australis*	00/08	nd	nd	30.3 ± 0.1	3.3 ± 0.0
	*Microcitrus* × *Eremocitrus* hybrid	00/07	nd	nd	31.0 ± 0.9	3.1 ± 0.3
	*E. glauca* × *C.* × *sinensis* hybrid	00/11	nd	nd	28.5 ± 0.4	3.8 ± 0.1
	*Eremocitrus* × *Microcitrus* hybrid	00/08	nd	nd	33.4 ± 0.1	2.4 ± 0.0

*^a^Freq., number of Las-positive scions (Ct ≤ 34.0)/total number of plants evaluated (with Las-positive rootstocks, Ct ≤ 34.0).*

*^b^Ct avg, cycle threshold average determined through detection of the 16S DNA by qPCR.*

*^c^SEM, standard error of the mean.*

*^d^Log, Las titer average in log10 of amplicon copies per gram of plant tissue estimated based on a standard curve as described by Lopes et al. (2013).*

^e^nd, non-detected.

*Rootstock bark avg ± SEM data from accessions in the category 2 were obtained exclusively from plants with positive (Ct ≤ 34.0) or suspicious to be positive (34.0 < Ct ≤ 36.0) leaves. Details on values of each plant are in the Supplementary Table 10.

The trigeneric hybrid ‘Faustrimedin,’ with half of its genome coming from *M. australasica*, showed only three scions out of 10 being Las-positive, but just at one time point and with Ct > 32.0 in the three cases. When re-evaluated at 24 MAI, four scions resulted to be Las-positive, having been three of them Las-negative until 12 MAI (Tables 2, 3 and Supplementary Tables 7, 10). *M. virgata*, another hybrid with half of *M. australasica* genome, showed three out 10 scions being Las-positive at 12 MAI. Only five of them could be re-evaluated at 24 MAI, being Las-negative, and the other five died due to Las-induced damages to the rootstock (Tables 2, 3 and Supplementary Tables 7, 10). *M. inodora* also was partially resistant, as six out of 13 scions resulted Las-positive at least at one time point, but as in the case of the *M. australasica* accessions and hybrids mentioned above, infections were inconsistent though bacterial titers were not so low as compared to those of the other genotypes. Las-induced damages in the rootstock killed two plants at 8 and 12 MAI and other two before 24 MAI, with three out of 10 scions resulting Las-positive. All accessions that were re-evaluated at 24 MAI had the bark tissue from the ‘Rangpur’ lime rootstocks also analyzed by qPCR and all were confirmed as Las-infected (Table 3 and Supplementary Table 10).

Regarding the cross-incompatible *Citrus* relatives within this category, *Citropsis gilletiana* had three plants out of 13 that were Las-positive at 12 MAI, and other three with Ct ranging between 34.8 and 35.7 at least at one time point. Likewise, five out of nine *Naringi crenulata* scions were Las-positive but only at 12 MAI, and the bacterial titers were usually low, with Ct > 30.0 (Table 2 and Supplementary Table 7). As in Category 1, HLB-like symptoms resembling mineral deficiencies appeared in infected scions likely due to the severe root loss caused by the bacterium, which precluded evaluating sensitivity to Las infection in the scion.

Contrast analysis among Category 2 accessions showed that differences observed in bacterial titers were not significant within the group (*p* < 0.08), but they were significant when comparing Category 1 and 2 (*p* < 0.001). Moreover, there were significant differences in bacterial titers when specifically comparing ‘Pera’ and ‘Tobias’ sweet orange controls versus Category 2 (*p* < 0.0377) and when comparing *P. trifoliata* versus Category 2 accessions (*p* < 0.001).

#### Category 3. Resistant

The seven accessions included into this group were *Microcitrus warburgiana*, *M. papuana*, *M. australis*, a *Microcitrus* sp. × *E. glauca* hybrid, *Eremocitrus glauca*, an eremorange hybrid (*E. glauca* × *Citrus* × *sinensis*), and an *E. glauca* × *Microcitrus* sp. hybrid. A variable number of plants from these accessions showed Las-infected ‘Rangpur’ lime rootstocks, confirming the partial success of Las inoculation (Supplementary Table 1), but none of the leaf samples from scions grafted on Las-positive rootstocks resulted positive for Las at 12 MAI (Table 2 and Supplementary Table 9). Because of this, they were classified as resistant to Las. Although *E. glauca* was graft-compatible with ‘Rangpur’ lime, its growth and development was generally poor, being this an intrinsic characteristic of the accession and not related to bud union problems (Bitters et al., 1964, 1969). The other six accessions were further evaluated at 24 MAI and the full resistance was confirmed for all of them (Table 3 and Supplementary Table 10). The eremorange, the *Microcitrus* sp. × *E. glauca* hybrid and the *E. glauca* × *Microcitrus* sp. hybrid evaluated in this work for the first time, showed the best graft-compatibility with ‘Rangpur’ lime as scion growth was vigorous. Considering their genetic backgrounds, all the seven accessions are probably sexually compatible with *Citrus*.

To confirm that these accessions were truly resistant, namely that there was vascular connection at the grafts and there was bacterial movement from rootstock to scions through the vascular system, bark tissue from each scion was evaluated by qPCR at 5 and 30 cm above the grafting propagation line and at the canopy (21–152 cm, depending on the accession) at 24 MAI to assess the presence of the bacterium. Las was detected in the scion bark close to the bud union (5 cm) in most plants but as sampling was performed farther from the grafting line, Las was found in very few plants at low concentration (30 cm) or was not detected (canopy) (Table 4 and Supplementary Table 11).

**TABLE 4:** ‘*Candidatus* Liberibacter asiaticus’ detection in scions from each plant of Category 3 accessions plus sweet orange controls. Bark samples were taken at different distances from the rootstock (5 cm, 30 cm and at the canopy) at 24 months after inoculation.

Accession	Freq.^a^	05 cm		Freq.	30 cm		Freq.	Canopy (21–152 cm)	
		Ct avg^b^ ± SEM^c^	Log avg^d^ ± SEM		Ct avg ± SEM	Log avg ± SEM		Ct avg ± SEM	Log avg ± SEM
*Citrus* × *sinensis* ‘Pera’	41/41	25.5 ± 0.4	4.8 ± 0.1	41/41	25.4 ± 0.3	4.8 ± 0.1	41/41	25.0 ± 0.3	4.8 ± 0.1
*Citrus* × *sinensis* ‘Tobias’	09/09	26.7 ± 1.1	4.4 ± 0.33	09/09	25.2 ± 0.9	4.8 ± 0.3	09/09	23.8 ± 0.6	5.3 ± 0.2
*Microcitrus warbugiana*	04/06	31.7 ± 0.6	2.9 ± 0.2	00/06	nd^e^	nd	00/06	nd	nd
*Microcitrus papuana*	02/04	32.5 ± 0.4	2.6 ± 0.1	00/04	nd	nd	00/04	nd	nd
*Microcitrus australis*	07/08	32.8 ± 0.6	2.6 ± 0.2	00/08	nd	nd	00/08	nd	nd
*Microcitrus* × *Eremocitrus* hybrid	06/07	30.8 ± 0.5	3.2 ± 0.1	03/07	27.8 ± 1.0	4.1 ± 0.3	00/07	nd	nd
*Eremocitrus glauca*	06/07	31.6 ± 0.8	2.9 ± 0.3	00/07	nd	nd	00/07	nd	nd
*E. glauca* × *C.* × *sinensis* hybrid	11/11	31.2 ± 0.4	3.0 ± 0.1	04/11	30.6 ± 0.4	3.2 ± 0.1	00/11	nd	nd
*Eremocitrus* × *Microcitrus* hybrid	08/08	32.1 ± 0.3	2.8 ± 0.1	00/08	nd	nd	00/08	nd	nd

*^a^Freq., number of Las-positive bark scion samples (Ct ≤ 34.0)/total number of plants evaluated (with Las-positive rootstocks, Ct ≤ 34.0).*

*^b^Ct avg, cycle threshold average determined through detection of the 16S DNA by qPCR.*

*^c^SEM, standard error of the mean.*

*^d^Log, Las titer average in log10 of amplicon copies per gram of plant tissue estimated based on a standard curve as described by Lopes et al. (2013). ^e^nd, non-detected.*

### Distribution of the Response to Las Categories According to Phylogeny

**FIGURE 4:**
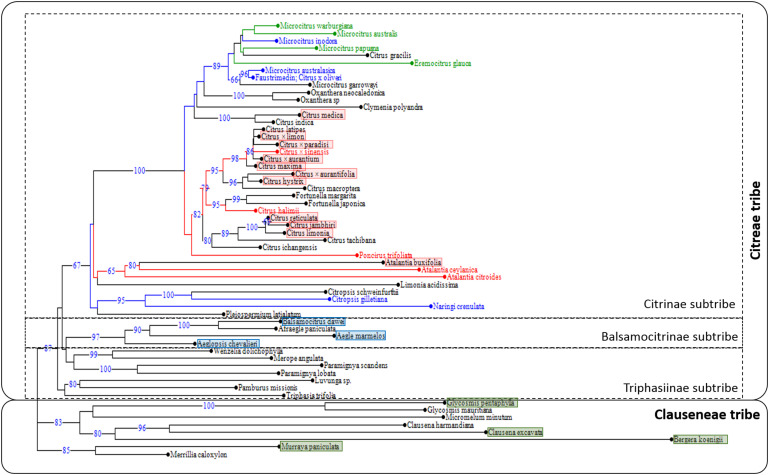
Distribution on the phylogenetic tree of the Aurantioideae sub-family (NJ tree based on eight chloroplast genome fragments) based on accession responses to Las challenge inoculation according to this study (red text: susceptible, blue text: partially resistant, green text: full resistant) and Ramadugu et al. (2016) (red rectangle: susceptible –categories 6 to 8; blue rectangle: tolerant –categories 3, 4; green rectangle: resistant –categories 1, 2).

A phylogenic tree was established using available data for eight genic and intergenic chloroplastic sequences (Bayer et al., 2009). Sequence alignment for the 59 accessions was performed with BioEdit software and manually curated for InDel regions. We identified 6430 positions with single nucleotide polymorphism and used them to establish the NJ tree (Figure 4). The True Citrus clade grouping the Oceanian genera *Microcitrus*, *Eremocitrus*, *Oxanthera* and *Clymenia* and the Asian genera *Citrus*, *Poncirus* and *Fortunella* was very well defined (bootstrap value = 100%). It was linked with lower support to a cluster joining three *Atalantia* species and *L. acidissima* and then a third cluster including two *Citropsis* species, *N. crenulata* and *Pleiospermium latialatum*. These three clusters constitute a clade quite well defined (bootstrap value = 67%) and differentiated from the other species of the Balsamocitrinae and Triphasiinae subtribes and the Clauseneae tribe (Figure 4).

Resistance to Las (category 1 here and categories 6 to 8 for Ramadugu et al., 2016) appeared to be concentrated in the clade joining *Atalantia* species with the True Citrus (Figure 4). Within this clade, there was a very strong phenotypic differentiation between the Oceanian species tested that were all classified as full resistant or partially resistant while all Asian citrus species analyzed here or previously assessed by Ramadugu et al. (2016) were found to be susceptible. In the Oceanian clade, all resistant species were grouped in a same sub-clade, differentiated (bootstrap value = 66) from another one grouping the partially resistant *M. australasica*, *C.* × *oliveri* and the non-tested *M. garrawayi*. Intriguingly, within the Citrinae subtribe, *C. gilletiana* and *N. crenulata* were partially resistant to Las (Figure 4). During their field evaluation under natural Las inoculation through *D. citri*, Ramadugu et al. (2016) found that all analyzed species of the Balsamocitrinae and Triphasiinae subtribes as well as those of Clauseneae tribe were resistant or tolerant to HLB (categories 1–2 and 3–4 of their study, respectively).

## Discussion

Searching for resistance to HLB within *Citrus* and its relatives of the family Rutaceae, subfamily Aurantioideae, has been an active area of research due to the severe damages caused by the disease on tree performance, production and fruit quality. Citriculture costs in HLB-affected regions have been increased due to the implementation of treatments to keep citrus groves economically productive but curative methods capable of overcoming losses are lacking (Bassanezi et al., 2020).

Therefore, there is a need for confident and reliable sources of resistance to either the Las bacterium, the insect vector *D. citri* or both, which could be used for introgression into the *Citrus* germplasm (i) to generate new *Citrus*-like cultivars that may be useful as rootstocks or scions, (ii) to map and identify the genes involved in the resistance trait for direct modification of well-known elite *Citrus* cultivars using modern biotechnology tools, especially those which cannot be improved by sexual breeding due to their high heterozygosity, or (iii) to be used promptly as new rootstocks or interstocks to potentially alleviate HLB-induced damages.

However, the characteristics of Las infection in *Citrus* genotypes, cultivars and relatives have generally rendered confounding results, mainly because symptoms appear many months after infection which delays disease development (Hung et al., 2001; Folimonova et al., 2009; Cifuentes-Arenas et al., 2019), they can be mistaken with those derived from nutritional deficiencies, at least at the beginning of infection (da Graça, 1991; Bové, 2006), the uneven distribution of the bacterium within the infected trees (Tatineni et al., 2008; Li et al., 2009; Raiol-Junior et al., 2021), its active multiplication quite restricted to new flushes and developing roots (Hilf and Luo, 2018; Raiol-Junior et al., 2021), the environmental influences on bacterial multiplication and plant colonization (Lopes et al., 2009; Gasparoto et al., 2012) and largely unknown plant host-pathogen molecular interactions which certainly may affect their outcome. To further complicate this interplay, the psyllid vector *D. citri* shows preference for specific colors and volatile compounds emitted by the host plants (Wenninger et al., 2009; Patt and Sétamou, 2010; Hall et al., 2011), and once settled it prefers to feed and reproduce on young shoots rather than mature leaves (Hall et al., 2016; Cifuentes-Arenas et al., 2018). Moreover, psyllids exhibit a clear preference for some hosts within Rutaceae, subfamily Aurantioideae (*Bergera*, *Murraya*) over others (*Citrus*), while some Aurantioideae species were reported as intermediate [such as *Glycosmis pentaphylla* (Retz.) DC., *Clausena harmandiana* (Pierre) Guillaumin and *Zanthoxylum ailanthoides* (L.)] and other *Citrus* relatives as highly resistant to the psyllid, such as certain *Poncirus trifoliata* accessions (Westbrook et al., 2011; Richardson and Hall, 2013; Hall et al., 2015; Felisberto et al., 2019). Furthermore, the use of seedlings, especially in the case of Aurantioideae relatives, introduces another factor of variation, not only due to the different morphological, development and physiological features of juvenile vs. mature plants, but more importantly because many *Citrus* relatives are monoembryonic, so propagation through seeds does not provide clonal plants but segregating progenies genetically different to the mother genotype. Taking all this into consideration and aiming to evaluate resistance to Las within *Citrus* relatives undoubtably, we decided to center our study on the bacterium-host interaction in a way avoiding any interference of the above mentioned factors.

The Citrinae genotypes of interest were first propagated clonally onto the ‘Rangpur’ lime rootstock and then inoculated by grafting well-controlled Las-infected wood onto the selected plants. However, this was not trivial. In addition to difficulties on grafting success for clonal propagation due to the genetic distances among some of the Citrinae species used and *Citrus*, problems in transmitting Las to them also occurred. Because of the irregular distribution of Las in plant tissues, use of symptomatic and qPCR-positive segments from donor plants are recommended for challenge inoculation. However, even with this, variation in Las transmission efficiencies averaging 70 to 90% within *Citrus*, *Fortunella* and *Poncirus* germplasm, have been found (Folimonova et al., 2009). In this work, we decided to use the Las-susceptible ‘Rangpur’ lime as rootstock because this allowed ascertaining which composite plants were actually infected by Las. Although graft-inoculation was performed both in the scion and in the rootstock, only those plants with infected root system were considered for further resistance evaluation, as in no case we detected a Las-positive scion on a ‘Rangpur’ lime rootstock free from Las. Moreover, this procedure precluded erroneous categorization of false-negative plants as resistant. Furthermore, the upward movement of Las from the rootstock to the scion 5 cm above the graft unions was confirmed in those accessions showing full-resistance to Las.

In more than 30 *Citrus, Poncirus* and *Fortunella* hosts tested, Folimonova et al. (2009) determined that Las was unevenly distributed, with higher titers of the bacterium found in symptomatic tissue. This erratic spread within a plant led to classification of *P. trifoliata* initially as resistant, but after a subsequent test it resulted to be susceptible, thus being results on infection of this genotype inconsistent. In our experiments, the four *P. trifoliata* accessions evaluated were categorized as susceptible as the bacterium readily multiplied in all propagations tested. The delay of about 2–4 months to reach 100% infection in *P. trifoliata* genotypes as compared to the two sweet orange cultivars used as controls, may be attributed to the deciduous nature of the former, which made them to flush much less frequently than most citrus types. However, Ramadugu et al. (2016) also tested two *P. trifoliata* accessions for resistance to HLB, in this case by psyllid-mediated natural infection under field conditions, and classified ‘Simmons’ as resistant and ‘Little-leaf’ as showing delayed infection. Notably, in other studies these two accessions were among the most resistant *Poncirus* ones to oviposition by *D. citri* (Westbrook et al., 2011; Richardson and Hall, 2013; Hall et al., 2015), so the field-resistance attributed to these two accessions may be explained by a lack of preference by the vector, especially when exposed mixed with other more preferred hosts, and perhaps not to bacterial resistance. In other works on *D. citri* biology, the four *Poncirus* accessions used here were considered partial resistant to *D. citri* as the female insects laid significantly fewer eggs than in sweet orange controls (Hall et al., 2015, 2017, 2019). Therefore, *P. trifoliata* has interest as a possible source of genetic resistance to *D. citri* rather than to Las. Nevertheless, other *P. trifoliata* accessions should be tested confidently for Las-resistance, especially those being widely used in rootstock breeding programs.

Ramadugu et al. (2016) showed in their field experiment that *Microcitrus* and *Eremocitrus* genera may be useful sources of resistance to HLB, though they could not clarify whether they were resistant to *D. citri* or to Las. Moreover, they observed variation in seedlings disease response within *Microcitrus* and transient infection in *E. glauca*, likely due to segregation as they used zygotic seedlings. We confirmed here using clonal plants that Las-resistance is widely spread within the germplasm of both genera. Remarkably, our results on response to Las challenge-inoculation separated the species from New Guinea, *E. glauca* and *M. australis* in the group of resistant genotypes while *M. inodora* and *M. australasica* were included in the category of partially resistant types. Response to Las of *M. australasica* accessions and hybrids reinforced generally its categorization as partially resistant, with the only exception of their hybrids with *E. glauca*, which were full-resistant. Interestingly, all *E. glauca* hybrids used in this study were fully resistant to Las, suggesting that the determinants of resistance in *E. glauca* may have dominant inheritance, which is particularly engaging for introgression breeding schemes. Therefore, *E. glauca* and its hybrids as well as *M. australis* may be the most indicated ones among the Australian limes as parents in breeding efforts for generating *Citrus*-like cultivars resistant to Las. Conversely, those *Microcitrus* species and hybrids presenting partial resistance to Las would be less indicated as they would probably confer incomplete resistance to their progenies.

Although sexual-compatibility with *Citrus* is restricted to some True Citrus fruit trees (Swingle and Reece, 1967; Bayer et al., 2009), graft-compatibility is widely reported, at least to most Citrinae genera (sensu Bayer et al., 2009, after Swingle and Reece, 1967). There are also reports of graft-compatibility of *Citrus* on *Clausena* (Bitters et al., 1964) and on *Murraya paniculata* (Swingle and Reece, 1967), which are farther relatives to *Citrus* and have been reported as resistant to Las (Ramadugu et al., 2016; Cifuentes-Arenas et al., 2019), but being suitable hosts for *D. citri* (Felisberto et al., 2019). However, *Citrus* grafts on *Clausena* and *Murraya* could be kept alive under greenhouse conditions but none of them seemed recommendable in a field situation due to poor bud unions and progressive incompatibility problems (Bitters et al., 1969). Other promising, HLB-resistant “Remote Citroid fruit trees” (Swingle and Reece, 1967), such as *Glycosmis*, are graft-uncongenial with *Citrus* (Bitters et al., 1964). Based on Bitters et al. (1964, 1969, 1977), our studies of graft-compatibility with *Citrus* and response to Las challenge inoculation were centered on Citrinae. Most species used were graft-compatible on ‘Rangpur’ lime, with the exception of *Limonia acidissima. L. acidissima* has been described as compatible with *Citrus* both as a scion and as a rootstock (Bitters et al., 1964; Swingle and Reece, 1967; Yoshida, 1996; Siebert et al., 2015), but its compatibility with ‘Rangpur’ lime was not previously tested. Our experience with *L. acidissima* exemplifies that close phylogenetic relations could be used as an approach to foresee graft-compatibility, but it does not always predict successful bud unions (Bitters et al., 1977). *C. halimii* showed another type of unexpected incompatibility, not derived from the scion-rootstock union with ‘Rangpur’ lime, but from the use of ‘Valencia’ sweet orange budwood to challenge-inoculate Las onto *C. halimii* scions. At the beginning, we associated the weak growth of propagations to the high sensitivity of *C. halimii* to Las infection, as indicated by Folimonova et al. (2009), but gum exudation started to appear around 11 MAI at sweet orange-*C. halimii* graft unions, irrespective of whether the grafted budwood was Las-infected or not. We included this *Citrus* type in our studies because it had displayed some resistance to HLB in the field experiments performed by Ramadugu et al. (2016), but it showed to be clearly susceptible in our challenges, as also indicated by Folimonova et al. (2009). Regarding graft-union problems, further experiments should be performed to attempt to reveal the causes of the incompatibilities. From the cross-incompatible, graft-compatible *Citrus* relatives tested for resistance to Las, the two *Atalantia* species used were susceptible, as already suggested for *Atalantia citroides* by Feng et al. (2015) using zygotic seedlings, while *Citropsis gilletiana* and *Naringi crenulata* were considered as partially resistant. According to Bitters et al. (1964), both genotypes may be excellent rootstocks for *Citrus*, but clearly, those cross-compatible *Citrus* relatives included in the full-resistant category offered the best alternatives to be tested as *Citrus* rootstocks and interstocks, particularly those showing excellent rootstock-scion compatibility and vigor in our studies, which were the *E. glauca* hybrids with *Microcitrus* and sweet orange.

Looking at the distribution of the different types of responses to Las challenge inoculation with a phylogenetic view, we found that susceptible accessions (category 1 here and categories 6 to 8 for Ramadugu et al., 2016) were concentrated in a clade joining True Citrus and *Atalantia* species plus *L. acidissima*. The sister position of *L. acidissima* and *Atalantia* species as well as the monophyly of True Citrus plus *Atalantia* species was previously described from chloroplast phylogeny by Pfeil and Crisp (2008) as well as Bayer et al. (2009), which conduced them to include *L. acidissima* in the Citrinae subtribe. The sister position of *Atalantia* clade with the True Citrus clade was also validated at nuclear level (Nagano et al., 2018). For the subsequent node of the phylogenetic tree, with a clade including *Citropsis* species, *N. crenulata* and *Pleiospermium latialatum*, the two species evaluated in our study were found partially resistant. Even lacking data of the response of Triphasiinae subtribe to Las challenge inoculation, it seemed that the determinants of susceptibility to Las, understood to mean efficient bacterial multiplication and colonization of the whole tree, appeared in an ancestor of the clade joining *Atalantia* and True Citrus species. All *Atalantia* species have Indian and south East Asia origin while *Citropsis* species are from Africa. It may be hypothesized that the Las susceptibility determinants arose in a common ancestor of *Atalantia* and True Citrus species, in India/South East Asia after the separation of *Citropsis* from Asian genera of the Citrinae subtribe. Pfeil and Crisp (2008) estimated that this separation occurred 12.9 My ago (with a quite large probability interval from 7.0 to 20.7 My).

The distribution of the categories of Las response to challenge inoculation in the True Citrus clade was also highly contrasting, with all Oceanian species considered as resistant or partially resistant while all Asian *Citrus* species were susceptible to Las (Folimonova et al., 2009; Ramadugu et al., 2016; this work), including *C. medica*, which is sister species of Oceanian species according to chloroplast phylogeny (Pfeil and Crisp, 2008; Bayer et al., 2009; Carbonell-Caballero et al., 2015; this work), but allied in a robust clade with *C. maxima* and *C. micrantha* for nuclear phylogeny, as concluded from nuclear gene sequencing (Garcia-Lor et al., 2013), restriction site-associated DNA sequencing (Nagano et al., 2018) and whole-genome sequencing (Wu et al., 2018). Based on chloroplastic phylogeny, Bayer et al. (2009) included *Microcitrus* and *Eremocitrus* within the group of True Citrus fruit trees (sensu Swingle and Reece, 1967), and proposed to include them in the genus *Citrus* following Mabberley (2004). The monophylly of the True Citrus species and the cross-compatibility between Oceanian and Asian citrus species are strong arguments sustaining this suggestion (Ollitrault et al., 2020). However, nuclear phylogeny clearly identified two sister clades within the True Citrus, one for the Asian species and the other for the Oceanian ones, with either full or partial resistance only present in the Oceanian clade (and observed for all Oceanian species tested). Under the hypothesis that Las susceptibility determinants were present but not fixed in the ancestral population of the True Citrus plus *Atalantia* clade, the differentiation between Australian and Asian sub-clades may result from a founder effect in the two geographic regions or/and genetic drift.

According to Swingle and Reece (1967), *Microcitrus* and *Eremocitrus* evolved from a common ancestor probably resembling *M. warburgiana*, which together with *M. papuana* are native to New Guinea. From such an ancestral form, one line of evolution produced the Australian round lime (*M. australis*), another line culminated in *M. inodora*, and a third line of evolution led to the Australian finger limes, *M. australasica*. On the other hand, *E. glauca* rapidly evolved from the common ancestor with marked xerophytic adaptations to the Australian deserts. The chloroplast phylogeny, with the monophylly of all the previously mentioned species, confirm their common origin but, as previously described by Bayer et al. (2009), we observed two main clades for the Australian and New Guinean species. The first one included all full resistant species (*M. warbugiana*, *M. australis, M. papuana* and *E. glauca*) plus the partially resistant *M. inodora* while the species and hybrids tested for the second clade were all partially resistant (*M. australasica*, its hybrids with *M. australis, M. virgata* and with ‘Calamondin,’ *C.* × *oliveri*). Considering the historical lack of interactions between HLB-associated pathogens/vectors and the citrus germplasm native to Australia/New Guinea, as they are still lacking in Australia and were detected for the first time with limited spread in Papua/New Guinea only in 2003 (EPPO Global Database, 2020), it may be speculated that resistance to Las in Australian limes is actually due to lack of functional susceptibility genes, likely derived from commonly inactive genetic loci among them.

In conclusion, these results demonstrate that there is consistent, complete and unequivocal resistance to Las, that is absence of bacterial multiplication, in a small group of *Citrus* relatives including *E. glauca* as well as its hybrids with *Citrus* and *Microcitrus* tested here, and also in some *Microcitrus* species, which may be used directly to be assessed as possible *Citrus* rootstocks/interstocks, to breed them with *Citrus* types to generate new *Citrus*-like cultivars and to map specific loci involved in the Las resistance (or lack of susceptibility) phenotype/s. Further studies on the interaction of the Las-resistant vs. susceptible genotypes with *D. citri* and with Las at molecular level would also help in understanding host-pathogen-vector interactions, identify effectors and metabolites prone to genetic modulation in *Citrus* and therefore get full profit of Citrinae genetic resources to produce new citrus cultivars resistant to this ravaging bacterium.

## Data Availability Statement

The datasets presented in this study can be found in online repositories. The names of the repository/repositories and accession number(s) can be found in the article/Supplementary Material.

## Author Contributions

LP and MA conceptualized and designed the work. MA and LR-J collected the data. MA, SL, LR-J, NW, EG, PO, and LP contributed to data analysis and interpretation, drafting the article and critical revision of the article. All authors contributed to the article and approved the submitted version.

## Conflict of Interest

The authors declare that the research was conducted in the absence of any commercial or financial relationships that could be construed as a potential conflict of interest.
