# Supplementary material for: Resistance to ‘Candidatus Liberibacter asiaticus,’ the Huanglongbing Associated Bacterium, in Sexually and/or Graft-Compatible Citrus Relatives
Source: Front Plant Sci. 2021 Jan 8;11:617664. doi: 10.3389/fpls.2020.617664 (PMC7820388; doi:10.3389/fpls.2020.617664)
Supplement: Supplementary file 1 [file Data_Sheet_1.docx]

Supplementary Material

**Supplementary Figure 1.** Standard curve generated using the amplified PCR product of the gene 16S rRNA from root of Las-positive samples.

**Supplementary Table 1.** List of SSR molecular markers used to assess parents from Australian lime hybrids.

|  |  |  |  |  |  |  |  |  |  |
| --- | --- | --- | --- | --- | --- | --- | --- | --- | --- |
|  | **Observed allelos** | | | | | | | **5' - 3' primer** | **Annealing Temperature (°C)** |
| **Marker name** | ***C. ×sinensis*** | ***E. glauca*** | ***M. autralasica*** | ***M. papuana*** | ***E. glauca* x *Microcitrus* sp.** | ***Microcitrus* sp*.* x *E. glauca*** | ***E. glauca* x *C. ×sinensis*** |  |  |
| Ci02B10 | 186 | 195-197-199 | 193-201-203 | 193-203 | 193-195-197 | 195-197-203 | 186-199 | F: TTTCACAGCCATCACA | 56 |
|  |  |  |  |  |  |  |  | R: AACACCAAGAAGGAAGAG |  |
| Ci07D10 | 151-153-154 | 142-145-146 | 141-143-145 | 145 | 142-143-145 | 142-143 | 145-146-151-153 | F: CGAGACAGACACAACAAAAA | 55 |
|  |  |  |  |  |  |  |  | R: AGAGGGTAATCCAAAAGACT |  |
| Ci01B10 | 158 | 145-167 | 145-157 | 145-157 | 145-167 | 145-150 | 158-167 | F: AAAAATTGCCCTCTTCTCCT | 50 |
|  |  |  |  |  |  |  |  | R: TGGTGGTTTTGTTGGTTCTAT |  |
| Ci01F08a | 114-135 | 109-115 | 105-110 | 106-110 | 105-109-110 | 106-109-110 | 109-114 | F: ATGAGCTAAAGAGAAGAGG | 50 |
|  |  |  |  |  |  |  |  | R: GGACTCAACACAACACAA |  |
| Ci07E05 | 109-113 | 111-113 | 104-108-109 | 104-108-109 | 109-111 | 104-108-109 | 108-111-113 | F: GGAGAACAAAACACAATG | 50 |
|  |  |  |  |  |  |  |  | R: ATCTTTCGGACAATCTT |  |
| MEST121 | 181-184 | 174-180 | 172-190 | 172-190 | 172-180 | 172-180 | 180-184 | F: TCCCTATCATCGGCAACTTC | 60 |
|  |  |  |  |  |  |  |  | R: CAATAATGTTAGGCTGGATGGA |  |
| Ci02A04 | 160-167 | 155-163 | 154-156-163 | 154-156-163 | 154-163 | 154-163 | 163-167 | F: CCGCTTTGTTCCATT | 55 |
|  |  |  |  |  |  |  |  | R: AGCGGTATCGTAATTCTC |  |
| Ci01G1 | 110 | 107-110-113 | 110-113 | 110-113 | 107-110-113 | 107-110-113 | 107-110 | F: ACTGTTGCTGCTGCTGCTGCT | 60 |
|  |  |  |  |  |  |  |  | R: TCGCTTTCTTATTTCACACTCACC |  |
| Ci01E02 | 153-169 | 161 | 155-159 | 155-159 | 155-161 | 155-161 | 153-161 | F: TGAATGGTACGGGAAATGC | 60 |
|  |  |  |  |  |  |  |  | R: CAGGGTCGGTGGAGAGGAT |  |
| Ci07B09 | 192-194 | 199-201 | 190-192 | 190-192 | 190-192-199-201 | 190-192-199-201 | 192-194-201 | F: AAACTGGAGTGCTAAATCT | 60 |
|  |  |  |  |  |  |  |  | R: AAAGAAGTTAAAGAAAAAATG |  |
|  |  |  |  |  |  |  |  |  |  |

**Supplementary Table 2**. Number of composite plants from each Citrinae genotype grafted with ‘Valencia’ sweet orange budwood pieces either infected with ‘*Candidatus* Liberibacter asiaticus’ (Las) or healthy (control). Total number of composite plants with Las-positive roots and frequency of infection from each accession and treatment. Infection frequency was the number of composite plants with positive ‘Rangpur’ lime roots X 100/total number of plants.

| **Genotype/Accession** | **Treatment** | **Number of plants used** | **Number of plants with Las-positive roots** | **Las-Infection Frequency (%)** |
| --- | --- | --- | --- | --- |
|  |  |  |  |  |
| *Citrus ×sinensis* ‘Pera’ | Las | 41 | 41 | 100 |
|  | Control | 33 | 0 | 0 |
| *C. ×sinensis* ‘Tobias’ | Las | 10 | 9 | 90 |
|  | Control | 5 | 0 | 0 |
| *C. halimii* | Las | 10 | 9 | 90 |
|  | Control | 9 | 0 | 0 |
| *Poncirus trifoliata*  ‘Pomeroy’ | Las | 8 | 6 | 75 |
|  | Control | 4 | 0 | 0 |
| *P. trifoliata*  ‘Benecke’ | Las | 8 | 6 | 75 |
|  | Control | 4 | 0 | 0 |
| *P. trifoliata* ‘Barnes’ | Las | 8 | 8 | 100 |
|  | Control | 4 | 0 | 0 |
| *P. trifoliata* ‘Rubidoux’ | Las | 8 | 6 | 75 |
|  | Control | 4 | 0 | 0 |
| *Microcitrus australasica* | Las | 12 | 11 | 92 |
|  | Control | 8 | 0 | 0 |
| *M. australasica* ‘Sanguinea’ | Las | 11 | 9 | 82 |
|  | Control | 10 | 0 | 0 |
| *M. australasica* ‘True Sanguinea’ | Las | 15 | 11 | 73 |
|  | Control | 10 | 0 | 0 |
| ‘Faustrimedin’ hybrid; *C*. x *oliveri* | Las | 10 | 10 | 100 |
|  | Control | 4 | 0 | 0 |
| *Microcitrus inodora* | Las | 15 | 13 | 87 |
|  | Control | 14 | 0 | 0 |
| *Microcitrus warburgiana* | Las | 12 | 9 | 75 |
|  | Control | 12 | 0 | 0 |
| *Microcitrus papuana* | Las | 10 | 8 | 80 |
|  | Control | 4 | 0 | 0 |
| *Microcitrus australis* | Las | 16 | 10 | 63 |
|  | Control | 13 | 0 | 0 |
| *Microcitrus virgata* hybrid | Las | 13 | 10 | 77 |
|  | Control | 11 | 0 | 0 |
| *Microcitrus* sp. x *Eremocitrus glauca* hybrid | Las | 10 | 7 | 70 |
|  | Control | 6 | 0 | 0 |
| *Eremocitrus glauca* | Las | 7 | 7 | 100 |
|  | Control | 4 | 0 | 0 |
| *E. glauca* x *C. ×sinensis* hybrid | Las | 13 | 12 | 92 |
|  | Control | 13 | 0 | 0 |
| *E. glauca* x *Microcitrus* sp. hybrid | Las | 10 | 8 | 80 |
|  | Control | 10 | 0 | 0 |
| *Atalantia citroides* | Las | 12 | 11 | 92 |
|  | Control | 8 | 0 | 0 |
| *Atalantia ceylanica* | Las | 12 | 12 | 100 |
|  | Control | 8 | 0 | 0 |
| *Citropsis gilletiana* | Las | 13 | 13 | 100 |
|  | Control | 10 | 0 | 0 |
| *Naringi crenulata* | Las | 10 | 9 | 90 |
|  | Control | 8 | 0 | 0 |

**Supplementary Table 3.** Access number of the Actin and Rubisco small subunit genes and the specific species used for the design of primers used as internal controls for qPCR of Citrinae roots.

| **GenBank** | **Number of Access** | **Specie** |
| --- | --- | --- |
| **Actin** | | |
| Phytozome | Ciclev10031847m | *Citrus ×clementina* |
| Phytozome | orange1.1g017102m | *Citrus ×sinensis* |
| Phytozome | AT3G53750.1 | *Arabidopsis thaliana* |
| Phytozome | GSVIVT01016550001 | *Vitis vinifera* |
| Phytozome | GRMZM2G082484_T01 | *Zea mays* |
| Phytozome | Solyc11g065990.1 | *Solanum lycopersicum* |
| **Rubisco small subunit** | | |
| Phytozome | Ciclev10009532m | *Citrus ×clementina* |
| Phytozome | Orange1.1g029245m | *Citrus ×sinensis* |
| Phytozome | Ciclev10012893m | *Zea mays* |
| NCBI | XM_010048675.1 | *Eucalyptus grandis* |
| NCBI | XM_004243031.3 | *Solanum lycopersicum* |
| NCBI | XR_531907.2 | *Malus domestica* |
| NCBI | XM_004296394.2 | *Fragaria vesca* |
| NCBI | XM_002270081.3 | *Vitis vinifera* |
| NCBI | XM_021419980.1 | *Herrania umbratica* |

**Supplementary Table 4.** Sequence of the primers designed and used as internal controls, qPCR conditions and reagents concentration.

| **Target sequence** | **Foward and Reverse primer names** | **5' - 3' primer sequence** | **Temperature (°C) and durations (sec) of denaturation, annealing and extension and total number of cycles** | **Reagents concentration** |
| --- | --- | --- | --- | --- |
| Actin | Actin_Forward | GGCATCACACTTTTTACAATG | 50°C, 2 min, 95°C, 10 min, 45°C, 15 s, 60°C, 40 s, 40 | TaqMan® PCR Master Mix Applied Biosystems (1x), 3,0 μM of each primer, 1 μL of gDNA at 100 ηg/μL, Milli-Q water q.s.p. |
|  | Actin_Reverse | TCAAACATGATCTGGGTCATC |  |  |
| Rubisco small subunit | Rubisco_Forward | CCATTCAATATCAAAACCTGC |  |  |
|  | Rubisco_Reverse | GGATGGGTGTACCGTGAG |  |  |

**Supplementary Table 5.** List of species used to establish the Aurantioideae phylogenic tree with the corresponding sequence accessions (GenBank) of the eight chloroplastic regions used.

| **Tribe** | **Sub-tribe** | | **Genus** | **Species** | **Reference of sequence accession (GenBank)** | | | | | | | |
| --- | --- | --- | --- | --- | --- | --- | --- | --- | --- | --- | --- | --- |
|  |  |  |  |  | **atpB-coding region** | **rbcL-atpB spacer** | **matK-5'trnK spacer** | **psbM-trnDGUC spacer** | **trnG intron** | **rps16 spacer** | **trnL-F region** | **rps4-trnT spacer** |
| Clauseneae | Micromelinae (very remote citroid fruit trees) | | *Micromelum* | *Micromelum minutum* | EF118889 | AF320854 | EF138904 | EF164876 | EF176560 | AF320266 | EF126691 | EF134696 |
|  | Clauseninae (remote citroid fruit trees) | | *Clausena* | *Clausena excavata* | AF066841 | AF320849 | EF138881 | EF164853 | EF176537 | AY295258 | EF126674 | EF134673 |
|  |  |  |  | *Clausena harmandiana* | EF118868 | AF320892 | EF138882 | EF164854 | EF176538 | EF126608 | EF126675 | EF134674 |
|  |  |  | *Glycosmis* | *Glycosmis mauritiana* | EF118878 | AF320862 | EF138892 | EF164864 | EF176548 | EF126614 | EF126681 | EF134684 |
|  |  |  |  | *Glycosmis pentaphylla* | EF118877 | EF126548 | EF138893 | EF164865 | EF176549 | EF126615 | EF126682 | EF134685 |
|  |  |  | *Murraya* | *Bergera koenigii* | EF118832 | AF320867 | EF138843 | EF164815 | EF176499 | AF320262 | EF126637 | EF134635 |
|  |  |  |  | *Murraya paniculata* | EF118891 | AF320868 | EF138906 | EF164878 | EF176562 | AY295254 | AY295280 | EF134698 |
|  | Merrilliinae (large-fruited remote citroid fruit trees) | | *Merrillia* | *Merrillia caloxylon* | EF118882 | AF320871 | EF138897 | EF164869 | EF176553 | AY295270 | AY295296 | EF134689 |
| Citreae | Triphasiinae (minor citroid fruit trees) | | *Luvunga* | *Luvunga sp.* | EF118880 | EF126549 | EF138895 | EF164867 | EF176551 | EF126617 | EF126684 | EF134687 |
|  |  |  | *Merope* | *Merope angulata* | EF118881 | EF126550 | EF138896 | EF164868 | EF176552 | EF126618 | EF126685 | EF134688 |
|  |  |  | *Oxanthera* | *Oxanthera neocaledonica* | EF118893 | EF126559 | EF138908 | EF164880 | EF176564 | EF126625 | EF126693 | EF134700 |
|  |  |  |  | *Oxanthera sp* | EF118894 | EF126560 | EF138909 | EF164881 | EF176565 | EF126626 | EF126694 | EF134701 |
|  |  |  | *Pamburus* | *Pamburus missionis* | EF118895 | AF320883 | EF138910 | EF164882 | EF176566 | AY295274 | AY295300 | EF134702 |
|  |  |  | *Paramignya* | *Paramignya lobata* | EF118896 | EF126561 | EF138911 | EF164883 | EF176567 | EF126627 | EF126695 | EF134703 |
|  |  |  |  | *Paramignya scandens* | EF118897 | EF126562 | EF138912 | EF164884 | EF176568 | AY295257 | EF126696 | EF134704 |
|  |  |  | *Triphasia* | *Triphasia trifolia* | EF118902 | AF320884 | EF138921 | EF164893 | EF176577 | AY295271 | AY295297 | EF134713 |
|  |  |  | *Wenzelia* | *Wenzelia dolichophylla* | EF118903 | EF126566 | EF138922 | EF164894 | EF176578 | AY295260 | AY295286 | EF134714 |
|  | Balsamocitrinae (hard-shelled citroid fruit trees) | | *Aegle* | *Aegle marmelos* | AF066839 | AF320882 | EF138836 | EF164808 | EF176492 | AY295268 | AY295294 | EF134628 |
|  |  |  | *Aeglopsis* | *Aeglopsis chevalieri* | EF118827 | EF126500 | EF138837 | EF164809 | EF176493 | EF126567 | EF126634 | EF134629 |
|  |  |  | *Afraegle* | *Afraegle paniculata* | EF118828 | EF126501 | EF138838 | EF164810 | EF176494 | AY295269 | AY295295 | EF134630 |
|  |  |  | *Balsamocitrus* | *Balsamocitrus dawei* | EF118831 | EF126504 | EF138842 | EF164814 | EF176498 | EF126571 | AY295278 | EF134634 |
|  |  |  | *Limonia* | *Limonia acidissima* | EF118870 | EF126541 | EF138885 | EF164857 | EF176541 | EF126609 | EF126676 | EF134677 |
|  | Citrinae (citrus fruit trees) | Near Citrus Fruit | *Atalantia* | *Atalantia ceylanica* | AF066840 | EF126502 | EF138839 | EF164811 | EF176495 | EF126568 | AY295288 | EF134631 |
|  |  |  |  | *Atalantia buxifolia* | AF066835 | AF320886 | EF138916 | EF164888 | EF176572 | EF126629 | AY295290 | EF134708 |
|  |  |  |  | *Atalantia citroides* | EF118829 | EF126503 | EF138840 | EF164812 | EF176496 | EF126569 | EF126635 | EF134632 |
|  |  |  | *Citropsis* | *Citropsis daweana* | EF118837 | EF126509 | EF138848 | EF164820 | EF176504 | EF126576 | EF126642 | EF134640 |
|  |  |  |  | *Citropsis schweinfurthii* | EF118838 | EF126510 | EF138849 | EF164821 | EF176505 | EF126577 | EF126643 | EF134641 |
|  |  | Primitive Citrus Fruit | *Hesperethusa* | *Naringi crenulata* | EF118892 | EF126558 | EF138907 | EF164879 | EF176563 | AY295272 | AY295298 | EF134699 |
|  |  |  | *Pleiospermium* | *Pleiospermium latialatum* | EF118898 | EF126563 | EF138913 | EF164885 | EF176569 | EF126628 | EF126697 | EF134705 |
|  |  | True Citrus fruit | *Citrus* | *Citrus maxima* | EF118839 | EF126529 | EF138869 | EF164841 | EF176525 | EF126597 | EF126663 | EF134661 |
|  |  |  |  | *Citrus medica* | EF118860 | EF126531 | EF138871 | EF164843 | EF176527 | EF126599 | EF126665 | EF134663 |
|  |  |  |  | *Citrus ichangensis* | EF118849 | EF126519 | EF138859 | EF164831 | EF176515 | EF126587 | EF126653 | EF134651 |
|  |  |  |  | *Citrus reticulata* | EF118864 | EF126537 | EF138877 | EF164849 | EF176533 | EF126604 | EF126670 | EF134669 |
|  |  |  |  | *Citrus ×aurantifolia* | EF118841 | AF320875 | EF138851 | EF164823 | EF176507 | EF126579 | EF126645 | EF134643 |
|  |  |  |  | *Citrus ×aurantium* | EF118842 | EF126512 | EF138852 | EF164824 | EF176508 | EF126580 | EF126646 | EF134645 |
|  |  |  |  | *Citrus ×limonia* | EF118856 | EF126526 | EF138866 | EF164838 | EF176522 | EF126594 | EF126660 | EF134658 |
|  |  |  |  | *Citrus ×limon* | EF118855 | EF126525 | EF138865 | EF164837 | EF176521 | EF126593 | EF126659 | EF134657 |
|  |  |  |  | *Citrus ×jambhiri* | EF118851 | EF126521 | EF138861 | EF164833 | EF176517 | EF126589 | EF126655 | EF134653 |
|  |  |  |  | *Citrus ×paradisi* | AJ238408 | EF126534 | EF138874 | EF164846 | EF176530 | AY295251 | AY295277 | EF134666 |
|  |  |  |  | *Citrus ×sinensis* | EF118866 | EF126539 | EF138879 | EF164851 | EF176535 | EF126606 | EF126672 | EF134671 |
|  |  |  |  | *Citrus halimii* | EF118847 | EF126517 | EF138857 | EF164829 | EF176513 | EF126585 | EF126651 | EF134649 |
|  |  |  |  | *Citrus hystrix* | EF118848 | EF126518 | EF138858 | EF164830 | EF176514 | EF126586 | EF126652 | EF134650 |
|  |  |  |  | *Citrus macroptera* | EF118857 | EF126527 | EF138867 | EF164839 | EF176523 | EF126595 | EF126661 | EF134659 |
|  |  |  |  | *Citrus indica* | EF118850 | EF126520 | EF138860 | EF164832 | EF176516 | EF126588 | EF126654 | EF134652 |
|  |  |  |  | *Citrus latipes* | EF118854 | EF126524 | EF138864 | EF164836 | EF176520 | EF126592 | EF126658 | EF134656 |
|  |  |  |  | *Citrus tachibana* | EF118867 | EF126540 | EF138880 | EF164852 | EF176536 | EF126607 | EF126673 | EF134672 |
|  |  |  |  | *Citrus gracilis* | EF118846.1 | EF126516.1 | EF138856.1 | EF164828.1 | EF176512.1 | EF126584.1 | EF126650.1 | EF134648 |
|  |  |  |  | Faustrimedin (*Citrus x oliveri)* | EF118876.1 | EF126547.1 | EF138891.1 | EF164863.1 | EF176547.1 | EF126613.1 | EF126678.1 | EF134683 |
|  |  |  | *Clymenia* | *Clymenia polyandra* | EF118869 | AF320878 | EF138883 | EF164855 | EF176539 | AY295255 | AY295281 | EF134675 |
|  |  |  | *Eremocitrus* | *Eremocitrus glauca* | AF066847 | AF320879 | EF138884 | EF164856 | EF176540 | AY295267 | AY295293 | EF134676 |
|  |  |  | *Fortunella* | *Fortunella japonica* | EF118873 | EF126544 | EF138888 | EF164860 | EF176544 | EF126611 | EF126679 | EF134680 |
|  |  |  |  | *Fortunella margarita* | EF118874 | EF126545 | EF138889 | EF164861 | EF176545 | EF126612 | EF126680 | EF134681 |
|  |  |  | *Microcitrus* | *Microcitrus australis* | EF118884 | EF126552 | EF138899 | EF164871 | EF176555 | EF126620 | EF126687 | EF134691 |
|  |  |  |  | *Microcitrus australasica* | EF118883 | EF126551 | EF138898 | EF164870 | EF176554 | EF126619 | EF126686 | EF134690 |
|  |  |  |  | *Microcitrus inodora* | EF118886 | EF126554 | EF138901 | EF164873 | EF176557 | EF126621 | EF126688 | EF134693 |
|  |  |  |  | *Microcitrus garrowayi* | EF118885 | EF126553 | EF138900 | EF164872 | EF176556 | AY295261 | AY295287 | EF134692 |
|  |  |  |  | *Microcitrus papuana* | EF118887 | EF126555 | EF138902 | EF164874 | EF176558 | EF126622 | EF126689 | EF134694 |
|  |  |  |  | *Microcitrus warburgiana* | EF118888 | EF126556 | EF138903 | EF164875 | EF176559 | EF126623 | EF126690 | EF134695 |
|  |  |  | *Poncirus* | *Poncirus trifoliata* | AJ238409 | AF320876 | EF138914 | EF164886 | EF176570 | AY295256 | AY295282 | EF134706 |

**Supplementary Table 6**. Time-course of ‘*Candidatus* Liberibacter asiaticus’ infection in the nine accessions included in Category 1, susceptible, as determined through detection of the bacterial 16S rDNA by qPCR.

| **Plant number** | **Scion** | | | | | | | | | |  | **Rootstock** | | | |
| --- | --- | --- | --- | --- | --- | --- | --- | --- | --- | --- | --- | --- | --- | --- | --- |
|  | **Leaves** | | | | | | | | | |  | **Root** | | **Bark** | |
|  | **4 MAI^a^** | | **6 MAI** | | **8 MAI** | | **10 MAI** | | **12 MAI** | |  | **12 MAI** | | | |
|  | **Ct^b^** | **Log^c^** | **Ct** | **Log** | **Ct** | **Log** | **Ct** | **Log** | **Ct** | **Log** |  | **Ct** | **Log** | **Ct** | **Log** |
| *Citrus ×sinensis* ‘Pera’/‘Rangpur’ lime | | | | | | | | | | | | | | | |
| 1 | 23.5 | 5.3 | 24.8 | 4.9 | 28.9 | 3.7 | 26.6 | 4.4 | 23.1 | 5.5 |  | 33.4 | 2.4 | 28.2 | 3.9 |
| 2 | 25.6 | 4.7 | 25.1 | 4.9 | 28.4 | 3.9 | 31.9 | 2.8 | 31.4 | 3.0 |  | 33.8 | 2.3 | 23.4 | 5.4 |
| 3 | nd^d^ | nd | nd | nd | nd | nd | 24.1 | 5.2 | 22.9 | 5.5 |  | 33.6 | 2.3 | 25.6 | 4.7 |
| 4 | 25.6 | 4.7 | 25.0 | 4.9 | 28.4 | 3.9 | 27.4 | 4.2 | 25.6 | 4.7 |  | 33.7 | 2.3 | 29.8 | 3.5 |
| 5 | 22.9 | 5.5 | 26.5 | 4.4 | 25.8 | 4.6 | 29.0 | 3.7 | 25.3 | 4.8 |  | 32.3 | 2.7 | 30.1 | 3.4 |
| 6 | 25.9 | 4.6 | 25.4 | 4.8 | 28.1 | 4.0 | 27.7 | 4.1 | 26.8 | 4.4 |  | 33.2 | 2.4 | 25.6 | 4.7 |
| 7 | 23.4 | 5.4 | 28.2 | 3.9 | 30.2 | 3.3 | 27.6 | 4.1 | 28.1 | 4.0 |  | 33.1 | 2.5 | 29.8 | 3.5 |
| 8 | 23.3 | 5.4 | 27.6 | 4.1 | 26.3 | 4.5 | 29.1 | 3.7 | 21.3 | 6.0 |  | 33.0 | 2.5 | 25.6 | 4.7 |
| 9 | 25.7 | 4.7 | 28.6 | 3.8 | 26.1 | 4.6 | 27.6 | 4.1 | 22.9 | 5.5 |  | 31.8 | 2.8 | 24.9 | 4.9 |
| 10 | 23.6 | 5.3 | 24.9 | 4.9 | 24.4 | 5.1 | 29.2 | 3.6 | 25.6 | 4.7 |  | 32.4 | 2.7 | 23.2 | 5.4 |
| 11 | 35.4 | 1.8 | 26.0 | 4.6 | 24.9 | 4.9 | 28.1 | 4.0 | 26.8 | 4.4 |  | 27.7 | 4.1 | 29.9 | 3.4 |
| 12 | nd | nd | nd | nd | 25.4 | 4.8 | 28.1 | 4.0 | 21.6 | 5.9 |  | 27.7 | 4.1 | 28.9 | 3.7 |
| 13 | 31.5 | 2.9 | 27.8 | 4.0 | 22.0 | 5.8 | 21.6 | 5.9 | 21.4 | 6.0 |  | 30.4 | 3.3 | 29.8 | 3.5 |
| 14 | 28.6 | 3.8 | 25.5 | 4.7 | 24.8 | 5.0 | 22.8 | 5.6 | 27.8 | 4.1 |  | 33.9 | 2.2 | 27.9 | 4.0 |
| 15 | 25.2 | 4.8 | nd | nd | 24.5 | 5.0 | 20.0 | 6.4 | 21.8 | 5.8 |  | 33.7 | 2.3 | 27.8 | 4.1 |
| 16 | 27.2 | 4.2 | 25.8 | 4.7 | 24.7 | 5.0 | 22.3 | 5.7 | 27.0 | 4.3 |  | 33.4 | 2.4 | ne^e^ | ne |
| 17 | nd | nd | nd | nd | 30.0 | 3.4 | 32.0 | 2.8 | 29.0 | 3.7 |  | 32.0 | 2.8 | ne | ne |
| 18 | 27.4 | 4.2 | 24.0 | 5.2 | 25.0 | 4.9 | 26.0 | 4.6 | 24.0 | 5.2 |  | 33.0 | 2.5 | ne | ne |
| 19 | nd | nd | nd | nd | 24.7 | 5.0 | 28.0 | 4.0 | 30.0 | 3.4 |  | 34.0 | 2.2 | ne | ne |
| 20 | nd | nd | nd | nd | nd | nd | 29.0 | 3.7 | 30.0 | 3.4 |  | 30.0 | 3.4 | ne | ne |
| 21 | nd | nd | nd | nd | 34.0 | 2.2 | 30.0 | 3.4 | 29.0 | 3.7 |  | 31.0 | 3.1 | ne | ne |
| 22 | nd | nd | nd | nd | nd | nd | 30.0 | 3.4 | 27.0 | 4.3 |  | 32.0 | 2.8 | ne | ne |
| 23 | nd | nd | 24.7 | 5.0 | 24.7 | 5.0 | 24.1 | 5.2 | 23.0 | 5.5 |  | 34.0 | 2.2 | ne | ne |
| 24 | nd | nd | nd | nd | 31.0 | 3.1 | 29.0 | 3.7 | 25.0 | 4.9 |  | 26.0 | 4.6 | ne | ne |
| 25 | nd | nd | 34.0 | 2.2 | 34.0 | 2.2 | 30.0 | 3.4 | 30.2 | 3.3 |  | 26.4 | 4.5 | ne | ne |
| 26 | nd | nd | nd | nd | nd | nd | nd | nd | 33.0 | 2.5 |  | 25.3 | 4.8 | ne | ne |
| 27 | nd | nd | 30.0 | 3.4 | 29.0 | 3.7 | 30.0 | 3.4 | 28.0 | 4.0 |  | 33.0 | 2.5 | ne | ne |
| 28 | nd | nd | 28.0 | 4.0 | 29.0 | 3.7 | 30.0 | 3.4 | 28.0 | 4.0 |  | 34.0 | 2.2 | ne | ne |
| 29 | nd | nd | 29.0 | 3.7 | 30.0 | 3.4 | 27.0 | 4.3 | 26.0 | 4.6 |  | 30.0 | 3.4 | ne | ne |
| 30 | nd | nd | 28.0 | 4.0 | 29.0 | 3.7 | 30.0 | 3.4 | 29.3 | 3.6 |  | 30.3 | 3.3 | ne | ne |
| 31 | nd | nd | 29.0 | 3.7 | 30.0 | 3.4 | 27.0 | 4.3 | 30.2 | 3.3 |  | 32.0 | 2.8 | ne | ne |
| 32 | nd | nd | 23.0 | 5.5 | 23.0 | 5.5 | 24.0 | 5.2 | 21.5 | 5.9 |  | 24.8 | 5.0 | ne | ne |
| 33 | 32.0 | 2.8 | 30.0 | 3.4 | 28.0 | 4.0 | 28.0 | 4.0 | 29.9 | 3.4 |  | 29.3 | 3.6 | ne | ne |
| 34 | nd | nd | 23.0 | 5.5 | 22.0 | 5.8 | 21.0 | 6.1 | 21.9 | 5.8 |  | 24.4 | 5.1 | ne | ne |
| 35 | 29.0 | 3.7 | 23.0 | 5.5 | 22.0 | 5.8 | 22.0 | 5.8 | 22.6 | 5.6 |  | 23.9 | 5.2 | ne | ne |
| 36 | 25.0 | 4.9 | 25.0 | 4.9 | 23.0 | 5.5 | 23.5 | 5.3 | 30.8 | 3.2 |  | 25.7 | 4.7 | ne | ne |
| 37 | nd | nd | nd | nd | 30.0 | 3.4 | 28.0 | 4.0 | 21.0 | 6.1 |  | 24.3 | 5.1 | ne | ne |
| 38 | 24.0 | 5.2 | 24.0 | 5.2 | 23.5 | 5.3 | 23.0 | 5.5 | 23.4 | 5.4 |  | 24.6 | 5.0 | ne | ne |
| 39 | nd | nd | nd | nd | nd | nd | 30.0 | 3.4 | 20.7 | 6.2 |  | 23.8 | 5.3 | ne | ne |
| 40 | nd | nd | 30.0 | 3.4 | 29.0 | 3.7 | 28.0 | 4.0 | 21.3 | 6.0 |  | 24.5 | 5.0 | ne | ne |
| 41 | 29.0 | 3.7 | 28.0 | 4.0 | 28.0 | 4.0 | 26.0 | 4.6 | 21.3 | 6.0 |  | 23.6 | 5.3 | ne | ne |
| *Citrus ×sinensis* ‘Tobias’/‘Rangpur’ lime | | | | | | | | | | | | | | | |
| 1 | nd | nd | 32.4 | 2.7 | 23.7 | 5.3 | 24.5 | 5.0 | 21.2 | 6.0 |  | 26.8 | 4.4 | 25.6 | 4.7 |
| 2 | 29.0 | 3.7 | nd | nd | 23.4 | 5.4 | 23.1 | 5.5 | 21.3 | 6.0 |  | 27.5 | 4.1 | 27.0 | 4.3 |
| 3 | 23.8 | 5.3 | 24.2 | 5.1 | 24.4 | 5.1 | 30.5 | 3.2 | 22.5 | 5.6 |  | 26.5 | 4.5 | 27.7 | 4.1 |
| 4 | 32.7 | 2.6 | nd | nd | 24.0 | 5.2 | 23.4 | 5.4 | 23.1 | 5.5 |  | 27.6 | 4.1 | 28.6 | 3.8 |
| 5 | nd | nd | nd | nd | 25.0 | 4.9 | 27.5 | 4.1 | 24.4 | 5.1 |  | 34.0 | 2.2 | 33.2 | 2.4 |
| 6 | 30.2 | 3.3 | 24.6 | 5.0 | 22.8 | 5.6 | 22.5 | 5.6 | 21.7 | 5.9 |  | 29.6 | 3.5 | 28.6 | 3.8 |
| 7 | nd | nd | nd | nd | 28.4 | 3.9 | 25.7 | 4.7 | 23.2 | 5.4 |  | 34.0 | 2.2 | 30.1 | 3.4 |
| 8 | nd | nd | nd | nd | 27.1 | 4.3 | 26.5 | 4.5 | 22.5 | 5.6 |  | 34.0 | 2.2 | 33.1 | 2.5 |
| 9 | nd | nd | 33.9 | 2.2 | 21.3 | 6.0 | 23.0 | 5.5 | 21.1 | 6.1 |  | 29.1 | 3.7 | 32.1 | 2.8 |
| *Citrus halimii/*‘Rangpur’ lime | | | | | | | | | | | | | | | |
| 1 | nd | nd | nd | nd | 29.0 | 3.7 | 27.4 | 4.2 | D^f^ | D |  | D | D | D | D |
| 2 | 28.6 | 3.8 | 30.6 | 3.2 | 26.7 | 4.4 | 27.0 | 4.3 | D | D |  | D | D | D | D |
| 3 | nd | nd | nd | nd | nd | nd | nd | nd | 25.0 | 4.9 |  | 31.1 | 3.1 | 30.1 | 3.4 |
| 4 | 31.3 | 3.0 | nd | nd | 30.0 | 3.4 | 30.3 | 3.3 | D | D |  | D | D | D | D |
| 5 | nd | nd | nd | nd | nd | nd | nd | nd | 32.0 | 2.8 |  | 32.2 | 2.7 | 30.3 | 3.3 |
| 6 | nd | nd | nd | nd | nd | nd | nd | nd | 31.0 | 3.1 |  | 26.8 | 4.4 | 29.9 | 3.4 |
| 7 | nd | nd | nd | nd | 27.0 | 4.3 | 30.3 | 3.3 | D | D |  | D | D | D | D |
| 8 | nd | nd | nd | nd | nd | nd | nd | nd | 33.0 | 2.5 |  | 34.0 | 2.2 | 32.3 | 2.7 |
| 9 | nd | nd | nd | nd | nd | nd | nd | nd | 29.0 | 3.7 |  | 33.0 | 2.5 | 30.2 | 3.3 |
| *Poncirus trifoliata* ‘Pomeroy’*/‘*Rangpur’ lime | | | | | | | | | | | | | | | |
| 1 | nd | nd | nd | nd | nd | nd | nd | nd | 23.0 | 5.5 |  | 27.3 | 4.2 | 31.0 | 3.1 |
| 2 | nd | nd | 30.8 | 3.2 | 29.0 | 3.7 | 34.8 | 2.0 | 29.7 | 3.5 |  | 26.1 | 4.6 | 29.9 | 3.4 |
| 3 | nd | nd | nd | nd | nd | nd | nd | nd | 33.0 | 2.5 |  | 34.0 | 2.2 | 31.3 | 3.0 |
| 4 | nd | nd | 31.8 | 2.8 | 32.3 | 2.7 | 34.0 | 2.2 | 28.5 | 3.8 |  | 33.9 | 2.2 | 32.8 | 2.6 |
| 5 | nd | nd | 27.3 | 4.2 | 26.6 | 4.4 | 25.4 | 4.8 | 27.5 | 4.1 |  | 28.0 | 4.0 | 30.1 | 3.4 |
| 6 | nd | nd | nd | nd | 28.2 | 3.9 | 28.4 | 3.9 | 25.9 | 4.6 |  | 34.0 | 2.2 | 30.8 | 3.2 |
| *Poncirus trifoliata* ‘Benecke’*/‘*Rangpur’ lime | | | | | | | | | | | | | | | |
| 1 | nd | nd | nd | nd | 29.8 | 3.4 | 29.2 | 3.6 | 30.2 | 3.3 |  | 27.1 | 4.3 | 27.0 | 4.3 |
| 2 | nd | nd | 34.0 | 2.2 | 26.9 | 4.3 | 27.9 | 4.0 | 33.4 | 2.4 |  | 34.0 | 2.2 | 31.8 | 2.9 |
| 3 | nd | nd | nd | nd | nd | nd | nd | nd | 33.0 | 2.5 |  | 26.6 | 4.4 | 29.9 | 3.4 |
| 4 | nd | nd | 31.3 | 3.0 | 29.5 | 3.6 | 28.0 | 4.0 | 27.7 | 4.1 |  | 29.8 | 3.5 | 31.9 | 2.8 |
| 5 | nd | nd | nd | nd | nd | nd | 32.9 | 2.5 | 29.3 | 3.6 |  | 34.0 | 2.2 | 28.7 | 3.8 |
| 6 | nd | nd | nd | nd | nd | nd | nd | nd | 33.5 | 2.3 |  | 26.9 | 4.3 | 27.9 | 4.0 |
| *Poncirus trifoliata* ‘Barnes’/*‘*Rangpur’ lime | | | | | | | | | | | | | | | |
| 1 | nd | nd | nd | nd | nd | nd | nd | nd | 34.0 | 2.2 |  | 30.6 | 3.2 | 28.7 | 3.8 |
| 2 | nd | nd | nd | nd | nd | nd | nd | nd | 33.7 | 2.3 |  | 27.6 | 4.1 | 27.6 | 4.1 |
| 3 | nd | nd | nd | nd | nd | nd | nd | nd | 33.2 | 2.4 |  | 28.3 | 3.9 | 24.9 | 4.9 |
| 4 | nd | nd | nd | nd | nd | nd | 29.4 | 3.6 | 30.3 | 3.3 |  | 34.0 | 2.2 | 32.9 | 2.5 |
| 5 | nd | nd | nd | nd | nd | nd | nd | nd | 34.0 | 2.2 |  | 28.5 | 3.8 | 27.6 | 4.1 |
| 6 | nd | nd | nd | nd | nd | nd | 33.3 | 2.4 | 25.1 | 4.9 |  | 32.1 | 2.8 | 30.9 | 3.1 |
| 7 | nd | nd | nd | nd | nd | nd | nd | nd | 32.4 | 2.7 |  | 34.0 | 2.2 | 33.7 | 2.3 |
| 8 | nd | nd | nd | nd | 34.0 | 2.2 | 33.0 | 2.5 | 33.4 | 2.4 |  | 32.9 | 2.5 | 30.1 | 3.4 |
| *Poncirus trifoliata* ‘Rubidoux’*/*‘Rangpur’ lime | | | | | | | | | | | | | | | |
| 1 | 28.1 | 4.0 | 23.1 | 5.5 | 26.0 | 4.6 | 24.4 | 5.1 | 23.9 | 5.2 |  | 29.0 | 3.7 | 28.4 | 3.9 |
| 2 | nd | nd | nd | nd | 28.0 | 4.0 | 26.0 | 4.6 | 24.0 | 5.2 |  | 32.4 | 2.7 | 31.8 | 2.9 |
| 3 | nd | nd | nd | nd | nd | nd | 25.4 | 4.8 | 24.3 | 5.1 |  | 27.5 | 4.1 | 27.1 | 4.3 |
| 4 | nd | nd | 25.3 | 4.8 | 26.2 | 4.5 | 27.7 | 4.1 | 26.4 | 4.5 |  | 32.4 | 2.7 | 30.8 | 3.2 |
| 5 | nd | nd | 28.1 | 4.0 | 23.5 | 5.3 | 26.0 | 4.6 | 25.5 | 4.7 |  | 28.4 | 3.9 | 26.8 | 4.4 |
| 6 | nd | nd | 34.9 | 1.9 | nd | nd | nd | nd | 33.0 | 2.5 |  | 34.0 | 2.2 | 31.5 | 2.9 |
| *Atalantia citroides/‘*Rangpur’ lime | | | | | | | | | | | | | | | |
| 1 | nd | nd | 29.4 | 3.6 | 27.8 | 4.1 | 26.0 | 4.6 | 27.5 | 4.1 |  | 25.4 | 4.8 | 25.2 | 4.8 |
| 2 | nd | nd | 27.8 | 4.1 | 25.8 | 4.7 | 27.8 | 4.0 | 28.1 | 4.0 |  | 22.5 | 5.6 | 25.1 | 4.9 |
| 3 | 35.6 | 1.7 | 29.9 | 3.4 | 27.5 | 4.1 | 29.8 | 3.5 | 30.8 | 3.1 |  | 25.0 | 4.9 | 26.1 | 4.5 |
| 4 | nd | nd | 28.4 | 3.9 | 30.7 | 3.2 | 27.7 | 4.1 | 27.1 | 4.3 |  | 25.4 | 4.8 | 25.5 | 4.7 |
| 5 | nd | nd | 29.2 | 3.6 | 27.7 | 4.1 | 28.3 | 3.9 | 27.6 | 4.1 |  | 24.9 | 4.9 | 26.8 | 4.4 |
| 6 | 27.0 | 4.3 | 29.7 | 3.5 | 28.3 | 3.9 | 29.7 | 3.5 | 28.5 | 3.8 |  | 25.3 | 4.8 | 24.8 | 5.0 |
| 7 | nd | nd | nd | nd | nd | nd | 33.2 | 2.4 | 33.0 | 2.5 |  | 24.3 | 5.1 | 25.2 | 4.8 |
| 8 | nd | nd | nd | nd | nd | nd | nd | nd | 33.6 | 2.3 |  | 32.7 | 2.6 | 31.2 | 3.0 |
| 9 | nd | nd | 30.3 | 3.3 | 29.2 | 3.6 | 30.0 | 3.4 | d | d |  | d | d | d | d |
| 10 | nd | nd | 30.0 | 3.4 | 30.3 | 3.3 | 30.2 | 3.3 | 32.0 | 2.8 |  | 30.0 | 3.4 | 26.8 | 4.4 |
| 11 | nd | nd | nd | nd | 29.5 | 3.5 | 28.2 | 3.9 | 31.2 | 3.0 |  | 24.3 | 5.1 | 25.6 | 4.7 |
| *Atalantia ceylanica/‘*Rangpur’ lime | | | | | | | | | | | | | | | |
| 1 | nd | nd | 31.9 | 2.8 | 33.5 | 2.3 | 27.7 | 4.1 | 22.9 | 5.5 |  | 24.9 | 4.9 | 24.2 | 5.1 |
| 2 | nd | nd | nd | nd | nd | nd | 30.3 | 3.3 | 32.7 | 2.6 |  | 24.4 | 5.1 | 27.7 | 4.1 |
| 3 | nd | nd | nd | nd | nd | nd | 32.6 | 2.6 | 33.8 | 2.3 |  | 24.6 | 5.0 | 23.7 | 5.3 |
| 4 | nd | nd | nd | nd | nd | nd | nd | nd | 32.9 | 2.5 |  | 24.7 | 5.0 | 24.9 | 4.9 |
| 5 | nd | nd | nd | nd | nd | nd | 30.5 | 3.2 | 25.4 | 4.8 |  | 24.6 | 5.0 | 26.6 | 4.4 |
| 6 | nd | nd | nd | nd | nd | nd | nd | nd | 31.1 | 3.1 |  | 25.2 | 4.8 | 25.2 | 4.8 |
| 7 | nd | nd | nd | nd | nd | nd | 22.7 | 5.6 | 24.8 | 5.0 |  | 27.8 | 4.1 | 25.5 | 4.7 |
| 8 | nd | nd | nd | nd | nd | nd | 30.4 | 3.3 | 32.8 | 2.5 |  | 25.2 | 4.8 | 25.5 | 4.7 |
| 9 | nd | nd | nd | nd | 23.6 | 5.3 | 22.7 | 5.6 | 24.4 | 5.1 |  | 27.7 | 4.1 | 24.9 | 4.9 |
| 10 | nd | nd | nd | nd | 32.7 | 2.6 | 27.5 | 4.1 | 26.3 | 4.5 |  | 26.0 | 4.6 | 25.8 | 4.7 |
| 11 | nd | nd | nd | nd | nd | nd | nd | nd | 31.4 | 3.0 |  | 27.9 | 4.0 | 33.0 | 2.5 |
| 12 | nd | nd | nd | nd | nd | nd | 33.3 | 2.4 | 34.0 | 2.2 |  | 26.5 | 4.4 | 25.4 | 4.8 |

^a^MAI: Months after inoculation.

^b^Ct: Cycle threshold.

^c^Log: Las titer in log10 of amplicon copies per gram of plant tissue estimated based on a standard curve as described by Lopes et al., 2013.

^d^nd: Non-detected.

^e^ne: Not evaluated.

^f^D: Plant died.

**Supplementary Table 7**. Time-course of ‘*Candidatus* Liberibacter asiaticus’ infection in the eight accessions included in Category 2, partially resistant, as determined through detection of the 16S rDNA by qPCR.

| **Category 2** | | | | | | | | | | | | | | | |
| --- | --- | --- | --- | --- | --- | --- | --- | --- | --- | --- | --- | --- | --- | --- | --- |
| **Plant number** | **Scion** | | | | | | | | | |  | **Rootstock** | | | |
|  | **Leaves** | | | | | | | | | |  | **Root** | | **Bark** | |
|  | **4 MAI^a^** | | **6 MAI** | | **8 MAI** | | **10 MAI** | | **12 MAI** | |  | **12 MAI** | | | |
|  | **Ct^b^** | **Log^c^** | **Ct** | **Log** | **Ct** | **Log** | **Ct** | **Log** | **Ct** | **Log** |  | **Ct** | **Log** | **Ct** | **Log** |
| *Microcitrus australasica/‘*Rangpur’ lime | | | | | | | | | | | | | | | |
| 1 | nd^d^ | nd | nd | nd | nd | nd | 35.9 | 1.6 | 33.2 | 2.4 |  | 28.5 | 3.8 | 30.1 | 3.4 |
| 2 | nd | nd | nd | nd | nd | nd | nd | nd | 30.8 | 3.1 |  | 28.2 | 3.9 | 30.7 | 3.2 |
| 3 | nd | nd | nd | nd | nd | nd | nd | nd | 33.4 | 2.4 |  | 27.0 | 4.3 | 30.4 | 3.3 |
| 4 | nd | nd | nd | nd | nd | nd | nd | nd | 29.6 | 3.5 |  | 30.1 | 3.4 | 33.2 | 2.4 |
| 5 | nd | nd | nd | nd | 33.1 | 2.5 | 35.8 | 1.7 | nd | nd |  | 27.8 | 4.0 | 32.7 | 2.6 |
| 6 | nd | nd | nd | nd | 25.9 | 4.6 | nd | nd | nd | nd |  | 26.9 | 4.3 | 32.8 | 2.6 |
| 7 | nd | nd | nd | nd | 20.9 | 6.1 | 32.9 | 2.5 | 30.2 | 3.3 |  | 26.1 | 4.6 | 29.9 | 3.4 |
| 8 | nd | nd | nd | nd | 32.1 | 2.8 | nd | nd | nd | nd |  | 25.6 | 4.7 | 32.5 | 2.6 |
| 9 | nd | nd | nd | nd | nd | nd | 32.0 | 2.8 | 32.3 | 2.7 |  | 26.3 | 4.5 | 30.6 | 3.2 |
| 10 | nd | nd | nd | nd | 20.0 | 6.4 | nd | nd | nd | nd |  | 27.6 | 4.1 | 31.1 | 3.1 |
| 11 | nd | nd | nd | nd | 29.1 | 3.7 | nd | nd | nd | nd |  | 27.3 | 4.2 | 32.4 | 2.7 |
| *Microcitrus australasica* ‘Sanguinea’*/‘*Rangpur’ lime | | | | | | | | | | | | | | | |
| 1 | nd | nd | nd | nd | nd | nd | nd | nd | nd | nd |  | 29.5 | 3.5 | 30.1 | 3.4 |
| 2 | 30.2 | 3.3 | nd | nd | nd | nd | 34.0 | 2.2 | 24.0 | 5.2 |  | 33.1 | 2.5 | 30.3 | 3.3 |
| 3 | 33.2 | 2.4 | 33.4 | 2.4 | 33.0 | 2.5 | 26.6 | 4.4 | 25.0 | 4.9 |  | 26.7 | 4.4 | 28.9 | 3.7 |
| 4 | nd | nd | nd | nd | nd | nd | nd | nd | nd | nd |  | 27.6 | 4.1 | 25.1 | 4.9 |
| 5 | nd | nd | nd | nd | nd | nd | nd | nd | nd | nd |  | 26.7 | 4.4 | 29.6 | 3.5 |
| 6 | nd | nd | nd | nd | nd | nd | nd | nd | nd | nd |  | 30.1 | 3.4 | 28.4 | 3.9 |
| 7 | nd | nd | nd | nd | nd | nd | 25.2 | 4.8 | 28.0 | 4.0 |  | 27.7 | 4.1 | 27.8 | 3.5 |
| 8 | nd | nd | nd | nd | nd | nd | nd | nd | nd | nd |  | 26.3 | 4.5 | 29.6 | 3.5 |
| 9 | nd | nd | nd | nd | nd | nd | 26.1 | 4.6 | 27.0 | 4.3 |  | 28.3 | 3.9 | 27.9 | 4.0 |
| *Microcitrus australasica* ‘True Sanguinea’*/‘*Rangpur’ lime | | | | | | | | | | | | | | | |
| 1 | nd | nd | nd | nd | nd | nd | nd | nd | nd | nd |  | 29.9 | 3.4 | 30.2 | 3.3 |
| 2 | nd | nd | nd | nd | nd | nd | nd | nd | nd | nd |  | 34.0 | 2.2 | 30.3 | 3.3 |
| 3 | nd | nd | nd | nd | nd | nd | 30.5 | 3.3 | 33.8 | 2.3 |  | 26.8 | 4.4 | 29.3 | 3.6 |
| 4 | nd | nd | nd | nd | nd | nd | nd | nd | nd | nd |  | 28.0 | 4.0 | 30.2 | 3.3 |
| 5 | nd | nd | nd | nd | nd | nd | nd | nd | nd | nd |  | 27.7 | 4.1 | 30.1 | 3.4 |
| 6 | nd | nd | nd | nd | nd | nd | nd | nd | nd | nd |  | 31.6 | 2.9 | 29.9 | 3.4 |
| 7 | nd | nd | nd | nd | nd | nd | nd | nd | nd | nd |  | 28.6 | 3.8 | 30.8 | 3.2 |
| 8 | nd | nd | nd | nd | nd | nd | nd | nd | nd | nd |  | 31.6 | 2.9 | 31.7 | 2.9 |
| 9 | nd | nd | nd | nd | nd | nd | nd | nd | nd | nd |  | 31.0 | 3.1 | 33.4 | 2.4 |
| 10 | nd | nd | nd | nd | nd | nd | nd | nd | nd | nd |  | 34.0 | 2.2 | 31.1 | 3.1 |
| 11 | nd | nd | nd | nd | nd | nd | nd | nd | nd | nd |  | 34.0 | 2.2 | 32.3 | 2.7 |
| ‘Faustrimedin’ hybrid; *C*. x *oliveri* /‘Rangpur’ lime | | | | | | | | | | | | | | | |
| 1 | nd | nd | nd | nd | nd | nd | nd | nd | nd | nd |  | 29.7 | 3.5 | 30.8 | 3.2 |
| 2 | nd | nd | nd | nd | nd | nd | nd | nd | 34.0 | 2.2 |  | 34.0 | 2.2 | 31.0 | 3.1 |
| 3 | nd | nd | nd | nd | nd | nd | nd | nd | 32.2 | 2.7 |  | 32.9 | 2.5 | 30.6 | 3.2 |
| 4 | nd | nd | nd | nd | nd | nd | nd | nd | nd | nd |  | 30.1 | 3.4 | 30.9 | 3.1 |
| 5 | nd | nd | nd | nd | nd | nd | nd | nd | nd | nd |  | 27.0 | 4.3 | 28.7 | 3.8 |
| 6 | nd | nd | nd | nd | nd | nd | nd | nd | nd | nd |  | 28.0 | 4.0 | 30.8 | 3.2 |
| 7 | nd | nd | nd | nd | nd | nd | nd | nd | nd | nd |  | 27.2 | 4.2 | 27.6 | 4.1 |
| 8 | nd | nd | nd | nd | 33.5 | 2.3 | nd | nd | nd | nd |  | 27.1 | 4.3 | 32.8 | 2.6 |
| 9 | nd | nd | nd | nd | nd | nd | nd | nd | nd | nd |  | 29.7 | 3.5 | 32.7 | 2.6 |
| 10 | nd | nd | nd | nd | nd | nd | nd | nd | nd | nd |  | 32.5 | 2.6 | 33.1 | 2.5 |
| *Microcitrus inodora/‘*Rangpur’ lime | | | | | | | | | | | | | | | |
| 1 | nd | nd | nd | nd | nd | nd | nd | nd | nd | nd |  | 31.0 | 3.1 | 33.2 | 2.4 |
| 2 | 25.9 | 4.6 | 24.9 | 4.9 | 23.6 | 5.3 | 21.5 | 6.0 | 21.4 | 6.0 |  | 32.7 | 2.6 | 28.9 | 3.7 |
| 3 | nd | nd | 27.1 | 4.3 | D^e^ | D | D | D | D | D |  | D | D | D | D |
| 4 | nd | nd | nd | nd | nd | nd | nd | nd | nd | nd |  | 32.2 | 2.7 | 32.5 | 2.6 |
| 5 | 26.5 | 4.5 | nd | nd | nd | nd | nd | nd | nd | nd |  | 34.0 | 2.2 | 33.0 | 2.5 |
| 6 | nd | nd | nd | nd | nd | nd | 29.1 | 3.7 | 34.0 | 2.2 |  | 27.3 | 4.2 | 25.4 | 4.8 |
| 7 | nd | nd | nd | nd | nd | nd | nd | nd | nd | nd |  | 30.5 | 3.2 | 27.9 | 4.0 |
| 8 | nd | nd | nd | nd | nd | nd | nd | nd | nd | nd |  | 33.4 | 2.4 | 32.5 | 2.6 |
| 9 | nd | nd | nd | nd | nd | nd | nd | nd | nd | nd |  | 33.3 | 2.4 | 28.4 | 3.9 |
| 10 | nd | nd | nd | nd | nd | nd | nd | nd | nd | nd |  | 33.0 | 2.5 | 27.5 | 4.1 |
| 11 | 23.6 | 5.3 | 23.0 | 5.5 | 22.2 | 5.7 | 27.4 | 4.2 | 21.8 | 5.8 |  | 33.0 | 2.5 | 29.9 | 3.4 |
| 12 | 24.2 | 5.1 | 21.3 | 6.0 | 21.7 | 5.9 | 22.1 | 5.8 | 24.3 | 5.1 |  | 33.4 | 2.4 | 27.8 | 4.1 |
| 13 | nd | nd | nd | nd | nd | nd | nd | nd | nd | nd |  | 32.5 | 2.6 | 30.1 | 3.4 |
| *Microcitrus virgata* hybrid/‘Rangpur’ lime | | | | | | | | | | | | | | | |
| 1 | nd | nd | nd | nd | nd | nd | nd | nd | nd | nd |  | 28.3 | 3.9 | 27.7 | 4.1 |
| 2 | nd | nd | nd | nd | nd | nd | nd | nd | nd | nd |  | 28.6 | 3.8 | 30.1 | 3.4 |
| 3 | nd | nd | nd | nd | nd | nd | nd | nd | 33.0 | 2.5 |  | 34.0 | 2.2 | 32.2 | 2.7 |
| 4 | nd | nd | nd | nd | nd | nd | nd | nd | nd | nd |  | 28.0 | 4.0 | 31.9 | 2.8 |
| 5 | nd | nd | nd | nd | 33.0 | 2.5 | nd | nd | 27.0 | 4.3 |  | 28.0 | 4.0 | 29.8 | 3.5 |
| 6 | nd | nd | nd | nd | nd | nd | nd | nd | nd | nd |  | 34.0 | 2.2 | 33.8 | 2.3 |
| 7 | nd | nd | nd | nd | nd | nd | nd | nd | 30.1 | 3.4 |  | 28.4 | 3.9 | 33.7 | 2.3 |
| 8 | nd | nd | nd | nd | nd | nd | nd | nd | nd | nd |  | 29.7 | 3.5 | 28.3 | 3.9 |
| 9 | nd | nd | nd | nd | nd | nd | nd | nd | nd | nd |  | 32.8 | 2.6 | 30.3 | 3.3 |
| 10 | nd | nd | nd | nd | nd | nd | nd | nd | nd | nd |  | 29.0 | 3.7 | 28.8 | 3.8 |
| *Citropsis guilletiana*/‘Rangpur’ lime | | | | | | | | | | | | | | | |
| 1 | nd | nd | nd | nd | nd | nd | nd | nd | nd | nd |  | 25.7 | 4.7 | 26.1 | 4.6 |
| 2 | nd | nd | nd | nd | nd | nd | nd | nd | nd | nd |  | 24.4 | 5.1 | 25.9 | 4.6 |
| 3 | 34.8 | 1.9 | nd | nd | nd | nd | nd | nd | nd | nd |  | 23.7 | 5.3 | 29.2 | 3.6 |
| 4 | nd | nd | nd | nd | nd | nd | nd | nd | 35.2 | 1.8 |  | 24.6 | 5.0 | 28.1 | 4.0 |
| 5 | nd | nd | 36.0 | 1.6 | nd | nd | nd | nd | 25.1 | 4.8 |  | 24.9 | 4.9 | 25.9 | 4.6 |
| 6 | 32.0 | 2.8 | 32.2 | 2.7 | 32.0 | 2.8 | nd | nd | 35.7 | 1.7 |  | 25.4 | 4.8 | 26.7 | 4.4 |
| 7 | nd | nd | nd | nd | nd | nd | nd | nd | nd | nd |  | 26.8 | 4.4 | 25.8 | 4.6 |
| 8 | nd | nd | nd | nd | nd | nd | nd | nd | nd | nd |  | 24.9 | 4.9 | 26.7 | 4.4 |
| 9 | nd | nd | nd | nd | nd | nd | nd | nd | nd | nd |  | 24.3 | 5.1 | 25.8 | 4.6 |
| 10 | nd | nd | nd | nd | nd | nd | nd | nd | nd | nd |  | 23.3 | 5.4 | 26.4 | 4.5 |
| 11 | nd | nd | nd | nd | nd | nd | nd | nd | 32.7 | 2.6 |  | 25.1 | 4.9 | 31.9 | 2.8 |
| 12 | nd | nd | nd | nd | nd | nd | nd | nd | 32.3 | 2.7 |  | 23.9 | 5.2 | 25.2 | 4.8 |
| 13 | nd | nd | nd | nd | nd | nd | nd | nd | nd | nd |  | 25.2 | 4.8 | 25.5 | 4.8 |
| *Naringi crenulata*/‘Rangpur’ lime | | | | | | | | | | | | | | | |
| 1 | nd | nd | nd | nd | nd | nd | nd | nd | 33.5 | 2.3 |  | 26.2 | 4.5 | 29.2 | 3.6 |
| 2 | nd | nd | nd | nd | nd | nd | nd | nd | 33.2 | 2.4 |  | 27.0 | 4.3 | 28.3 | 3.9 |
| 3 | nd | nd | nd | nd | nd | nd | nd | nd | 30.6 | 3.2 |  | 27.3 | 4.2 | 26.7 | 4.4 |
| 4 | nd | nd | nd | nd | nd | nd | nd | nd | 30.3 | 3.3 |  | 26.0 | 4.6 | 29.1 | 3.7 |
| 5 | nd | nd | nd | nd | nd | nd | nd | nd | nd | nd |  | 25.7 | 4.7 | 26.5 | 4.4 |
| 6 | nd | nd | nd | nd | nd | nd | nd | nd | nd | nd |  | 28.5 | 3.8 | 27.0 | 4.3 |
| 7 | nd | nd | nd | nd | nd | nd | nd | nd | nd | nd |  | 26.0 | 4.6 | 27.1 | 4.3 |
| 8 | nd | nd | nd | nd | nd | nd | nd | nd | 33.0 | 2.5 |  | 27.5 | 4.2 | 30.7 | 3.2 |
| 9 | nd | nd | nd | nd | nd | nd | nd | nd | nd | nd |  | 27.8 | 4.0 | 28.8 | 3.8 |

^a^MAI: Months after inoculation.

^b^Ct: Cycle threshold.

^c^Log: Las titer in log10 of amplicon copies per gram of plant tissue estimated based on a standard curve as described by Lopes et al., 2013.

^d^nd: Non-detected.

^e^D: Plant died.

**Supplementary Table 8**. Comparison of the average values of bacterial titer in the positive rootstocks in which scions were positive versus the negative ones in the accessions from Category 2.

|  | **Acession** | **Freq.^a^** | **Scion** | |  | **Rootstock** | | | | |
| --- | --- | --- | --- | --- | --- | --- | --- | --- | --- | --- |
|  |  |  | **Leaves** | |  | **Root** | |  | **Bark** | |
|  |  |  |  |  |  |  |  |  |  |  |
|  |  |  | **Ct avg^b^**  **±SEM^c^** | **Log avg^d^**  **±SEM** |  | **Ct avg**  **±SEM** | **Log avg**  **±SEM** |  | **Ct avg**  **±SEM** | **Log avg**  **±SEM** |
| **Positive scions/positive rootstock** | *Microcitrus australasica* | 06/11 | 31.6±0.7 | 2.9±0.2 |  | 27.7±0.6 | 4.1±0.2 |  | 30.8±0.5 | 3.2±0.2 |
|  | *M. australasica* 'Sanguinea' | 04/09 | 26.0±0.9 | 4.6±0.3 |  | 29.0±1.4 | 3.7±0.4 |  | 28.7±0.6 | 3.6±0.1 |
|  | *M. australasica* 'True Sanguinea' | 01/11 | 33.8±0.0 | 2.3±0.0 |  | 26.8±0.0 | 4.4±0.0 |  | 29.3±0.0 | 3.6±0.0 |
|  | Faustrimedin hybrid; *C*. ×*oliveri* | 02/10 | 33.1±0.9 | 2.5±0.3 |  | 33.5±0.6 | 2.4±0.2 |  | 30.8±0.2 | 3.2±0.1 |
|  | *Microcitrus inodora* | 04/12 | 25.4±2.9 | 4.8±0.9 |  | 31.6±1.4 | 2.9±0.4 |  | 28.0±1.0 | 4.0±0.3 |
|  | *Microcitrus virgata* hybrid | 03/10 | 30.0±1.7 | 3.4±0.5 |  | 30.1±1.9 | 3.4±0.6 |  | 31.9±1.1 | 2.8±0.4 |
|  | *Citropsis gilletiana* | 05/13 | 32.2±1.9 | 2.7±0.6 |  | 24.8±0.3 | 5.0±0.1 |  | 27.6±1.2 | 4.1±0.4 |
|  | *Naringi crenulata* | 05/09 | 32.1±0.7 | 2.7±0.2 |  | 26.8±0.3 | 4.4±0.1 |  | 28.8±0.7 | 3.8±0.2 |
| **Negative scions/positive rootstock** | *Microcitrus australasica* | 05/11 | nd^e^ | nd |  | 27.0±0.4 | 4.3±0.1 |  | 32.3±0.3 | 2.7±0.1 |
|  | *M. australasica* 'Sanguinea' | 05/09 | nd | nd |  | 28.0±0.8 | 4.0±0.2 |  | 28.6±0.9 | 3.8±0.3 |
|  | *M. australasica* 'True Sanguinea' | 10/11 | nd | nd |  | 31.0±0.8 | 3.1±0.2 |  | 31.0±0.4 | 3.1±0.1 |
|  | Faustrimedin hybrid; *C*. ×*oliveri* | 08/10 | nd | nd |  | 28.9±0.7 | 3.7±0.2 |  | 30.9±0.7 | 3.1±0.2 |
|  | *Microcitrus inodora* | 08/12 | nd | nd |  | 32.5±0.4 | 2.6±0.1 |  | 30.6±0.9 | 3.2±0.3 |
|  | *Microcitrus virgata* hybrid | 07/10 | nd | nd |  | 30.1±0.9 | 3.4±0.3 |  | 30.1±0.8 | 3.4±0.2 |
|  | *Citropsis gilletiana* | 08/13 | nd | nd |  | 24.8±0.4 | 5.0±0.1 |  | 26.4±0.4 | 4.5±0.1 |
|  | *Naringi crenulata* | 04/09 | nd | nd |  | 27.0±0.7 | 4.3±0.2 |  | 27.4±0.5 | 4.2±0.1 |

^a^Freq.: Number of Las-positive and Las-negative scions/total of plants evaluated (with Las-positive rootstocks at Ct ≤ 34.0).

^b^Ct avg: Cycle threshold average determined through the detection of the 16S DNA by qPCR.

^c^SEM: Standard Error of the Mean.

^d^Log: Las titer average in log10 of amplicon copies per gram of plant tissue estimated based on a standard curve as described by Lopes et al., 2013.

^e^nd: Non-detected.

**Supplementary Table 9**. Time-course of ‘*Candidatus* Liberibacter asiaticus’ infection in the seven accessions included in Category 3, full-resistant, as determined through detection of the 16S rDNA by qPCR.

| **Category 3** | | | | | | | | | | | | | | | |
| --- | --- | --- | --- | --- | --- | --- | --- | --- | --- | --- | --- | --- | --- | --- | --- |
| **Plant number** | **Scion** | | | | | | | | | |  | **Rootstock** | | | |
|  | **Leaves** | | | | | | | | | |  | **Root** | | **Bark** | |
|  | **4 MAI^a^** | | **6 MAI** | | **8 MAI** | | **10 MAI** | | **12 MAI** | |  | **12 MAI** | | | |
|  | **Ct^b^** | **Log^c^** | **Ct** | **Log** | **Ct** | **Log** | **Ct** | **Log** | **Ct** | **Log** |  | **Ct** | **Log** | **Ct** | **Log** |
| *Microcitrus warburgiana/‘*Rangpur’ lime | | | | | | | | | | | | | | | |
| 1 | nd^d^ | nd | nd | nd | nd | nd | nd | nd | nd | nd |  | 33.2 | 2.4 | 30.2 | 3.3 |
| 2 | nd | nd | nd | nd | nd | nd | nd | nd | nd | nd |  | 31.7 | 2.9 | 32.7 | 2.6 |
| 3 | nd | nd | nd | nd | nd | nd | nd | nd | nd | nd |  | 32.1 | 2.8 | 30.8 | 3.1 |
| 4 | nd | nd | nd | nd | nd | nd | nd | nd | nd | nd |  | 33.1 | 2.5 | 31.2 | 3.0 |
| 5 | nd | nd | nd | nd | nd | nd | nd | nd | nd | nd |  | 27.3 | 4.2 | 28.3 | 3.9 |
| 6 | nd | nd | nd | nd | nd | nd | nd | nd | nd | nd |  | 33.2 | 2.4 | 30.2 | 3.3 |
| 7 | nd | nd | nd | nd | nd | nd | nd | nd | nd | nd |  | 30.7 | 3.2 | 28.4 | 3.9 |
| 8 | nd | nd | nd | nd | 35.4 | 1.8 | nd | nd | nd | nd |  | 33.3 | 2.4 | 27.4 | 4.2 |
| 9 | nd | nd | nd | nd | nd | nd | nd | nd | nd | nd |  | 32.5 | 2.7 | 31.6 | 2.9 |
| *Microcitrus papuana/‘*Rangpur’ lime | | | | | | | | | | | | | | | |
| 1 | nd | nd | nd | nd | nd | nd | nd | nd | nd | nd |  | 29.0 | 3.7 | 30.1 | 3.4 |
| 2 | nd | nd | nd | nd | nd | nd | nd | nd | nd | nd |  | 26.2 | 4.5 | 30.6 | 3.2 |
| 3 | nd | nd | nd | nd | nd | nd | nd | nd | nd | nd |  | 30.7 | 3.2 | 30.2 | 3.3 |
| 4 | nd | nd | nd | nd | nd | nd | nd | nd | nd | nd |  | 29.0 | 3.7 | 30.5 | 3.3 |
| 5 | nd | nd | nd | nd | nd | nd | nd | nd | nd | nd |  | 28.1 | 4.0 | 29.9 | 3.4 |
| 6 | nd | nd | nd | nd | nd | nd | nd | nd | nd | nd |  | 33.9 | 2.2 | 32.1 | 2.8 |
| 7 | nd | nd | nd | nd | nd | nd | nd | nd | nd | nd |  | 28.3 | 3.9 | 27.5 | 4.1 |
| 8 | nd | nd | nd | nd | nd | nd | nd | nd | nd | nd |  | 27.5 | 4.1 | 29.3 | 3.6 |
| *Microcitrus australis/‘*Rangpur’ lime | | | | | | | | | | | | | | | |
| 1 | nd | nd | nd | nd | nd | nd | nd | nd | nd | nd |  | 34.0 | 2.2 | 30.1 | 3.4 |
| 2 | nd | nd | nd | nd | nd | nd | nd | nd | nd | nd |  | 29.5 | 3.6 | 33.2 | 2.4 |
| 3 | nd | nd | nd | nd | nd | nd | nd | nd | nd | nd |  | 30.8 | 3.2 | 30.1 | 3.4 |
| 4 | nd | nd | nd | nd | nd | nd | nd | nd | nd | nd |  | 31.3 | 3.0 | 31.3 | 3.0 |
| 5 | nd | nd | nd | nd | nd | nd | nd | nd | nd | nd |  | 34.0 | 2.2 | 34.0 | 2.2 |
| 6 | nd | nd | nd | nd | nd | nd | nd | nd | nd | nd |  | 33.0 | 2.5 | 27.1 | 4.3 |
| 7 | nd | nd | nd | nd | nd | nd | nd | nd | nd | nd |  | 32.0 | 2.8 | 33.8 | 2.3 |
| 8 | nd | nd | nd | nd | nd | nd | nd | nd | nd | nd |  | 33.9 | 2.2 | 29.9 | 3.4 |
| 9 | nd | nd | nd | nd | nd | nd | nd | nd | nd | nd |  | 33.1 | 2.5 | 30.0 | 3.4 |
| 10 | nd | nd | nd | nd | nd | nd | 34.8 | 2.0 | nd | nd |  | 33.4 | 2.4 | 27.8 | 4.1 |
| *Microcitrus* sp. x *Eremocitrus glauca* hybrid/‘Rangpur’ lime | | | | | | | | | | | | | | | |
| 1 | nd | nd | nd | nd | nd | nd | nd | nd | nd | nd |  | 32.5 | 2.6 | 30.0 | 3.4 |
| 2 | nd | nd | nd | nd | nd | nd | nd | nd | nd | nd |  | 30.1 | 3.4 | 27.8 | 4.1 |
| 3 | nd | nd | nd | nd | nd | nd | nd | nd | nd | nd |  | 33.3 | 2.4 | 32.1 | 2.8 |
| 4 | nd | nd | nd | nd | nd | nd | nd | nd | nd | nd |  | 33.5 | 2.3 | 30.8 | 3.2 |
| 5 | nd | nd | nd | nd | nd | nd | nd | nd | nd | nd |  | 28.9 | 3.7 | 28.3 | 3.9 |
| 6 | nd | nd | nd | nd | nd | nd | nd | nd | nd | nd |  | 33.3 | 2.4 | 29.3 | 3.6 |
| 7 | nd | nd | nd | nd | nd | nd | nd | nd | nd | nd |  | 31.0 | 3.1 | 27.3 | 4.2 |
| *Eremocitrus glauca/‘*Rangpur’ lime | | | | | | | | | | | | | | | |
| 1 | nd | nd | nd | nd | nd | nd | nd | nd | nd | nd |  | 24.9 | 4.9 | 27.9 | 4.0 |
| 2 | nd | nd | nd | nd | nd | nd | nd | nd | nd | nd |  | 24.9 | 4.9 | 26.8 | 4.4 |
| 3 | nd | nd | nd | nd | nd | nd | nd | nd | nd | nd |  | 26.2 | 4.5 | 30.3 | 3.3 |
| 4 | nd | nd | nd | nd | nd | nd | nd | nd | nd | nd |  | 24.3 | 5.1 | 28.9 | 3.7 |
| 5 | nd | nd | nd | nd | nd | nd | nd | nd | nd | nd |  | 26.0 | 4.6 | 31.7 | 2.9 |
| 6 | nd | nd | nd | nd | nd | nd | nd | nd | nd | nd |  | 26.0 | 4.6 | 32.4 | 2.7 |
| 7 | nd | nd | nd | nd | nd | nd | nd | nd | nd | nd |  | 24.5 | 5.1 | 30.8 | 3.2 |
| *Eremocitrus glauca* x *Citrus ×sinensis* hybrid*/’*Rangpur’ lime | | | | | | | | | | | | | | | |
| 1 | nd | nd | nd | nd | nd | nd | nd | nd | nd | nd |  | 30.1 | 3.4 | 29.5 | 3.5 |
| 2 | nd | nd | nd | nd | nd | nd | nd | nd | nd | nd |  | 30.2 | 3.3 | 26.8 | 4.4 |
| 3 | nd | nd | nd | nd | nd | nd | nd | nd | nd | nd |  | 30.0 | 3.4 | 28.9 | 3.7 |
| 4 | nd | nd | nd | nd | nd | nd | nd | nd | nd | nd |  | 31.8 | 2.9 | 27.1 | 4.3 |
| 5 | nd | nd | nd | nd | nd | nd | nd | nd | nd | nd |  | 32.6 | 2.6 | 28.2 | 3.9 |
| 6 | nd | nd | nd | nd | nd | nd | nd | nd | nd | nd |  | 28.2 | 3.9 | 27.3 | 4.2 |
| 7 | nd | nd | nd | nd | nd | nd | nd | nd | nd | nd |  | 28.9 | 3.7 | 31.3 | 3.0 |
| 8 | nd | nd | nd | nd | nd | nd | nd | nd | nd | nd |  | 30.0 | 3.4 | 31.2 | 3.0 |
| 9 | nd | nd | nd | nd | nd | nd | nd | nd | nd | nd |  | 33.3 | 2.4 | 27.1 | 4.3 |
| 10 | nd | nd | nd | nd | nd | nd | nd | nd | nd | nd |  | 31.7 | 2.9 | 29.8 | 3.5 |
| 11 | nd | nd | nd | nd | nd | nd | nd | nd | nd | nd |  | 31.7 | 2.9 | 28.9 | 3.7 |
| 12 | nd | nd | nd | nd | nd | nd | nd | nd | nd | nd |  | 29.8 | 3.5 | 30.1 | 3.4 |
| *Eremocitrus glauca* x *Microcitrus* sp. hybrid/‘Rangpur’ lime | | | | | | | | | | | | | | | |
| 1 | nd | nd | nd | nd | nd | nd | nd | nd | nd | nd |  | 33.0 | 2.5 | 30.2 | 3.3 |
| 2 | nd | nd | nd | nd | nd | nd | nd | nd | nd | nd |  | 33.3 | 2.4 | 32.2 | 2.7 |
| 3 | nd | nd | nd | nd | nd | nd | nd | nd | nd | nd |  | 33.6 | 2.3 | 31.6 | 2.9 |
| 4 | nd | nd | nd | nd | nd | nd | nd | nd | nd | nd |  | 31.8 | 2.8 | 30.8 | 3.2 |
| 5 | nd | nd | nd | nd | nd | nd | nd | nd | nd | nd |  | 32.3 | 2.7 | 32.3 | 2.7 |
| 6 | nd | nd | nd | nd | nd | nd | nd | nd | nd | nd |  | 33.5 | 2.3 | 30.4 | 3.3 |
| 7 | nd | nd | nd | nd | nd | nd | nd | nd | nd | nd |  | 34.0 | 2.2 | 31.2 | 3.0 |
| 8 | nd | nd | nd | nd | nd | nd | nd | nd | nd | nd |  | 33.2 | 2.4 | 30.1 | 3.4 |

^a^MAI: Months after inoculation.

^b^Ct: Cycle threshold.

^c^Log: Las titer in log10 of amplicon copies per gram of plant tissue estimated based on a standard curve as described by Lopes et al., 2013.

^d^nd: Non-detected.

**Supplementary Table 10.** ‘*Candidatus* Liberibacter asiaticus’ infection in the Citrinae genotypes re-evaluated at 24 months after inoculation (MAI), as determined through detection of the 16S rDNA by qPCR.

| **Plant number** | **24 MAI** | | | |
| --- | --- | --- | --- | --- |
|  | **Scion Leaves** | | **Rootstock Bark** | |
|  | **Ct^a^** | **Log^b^** | **Ct** | **Log** |
| *Citrus* ×*sinensis* ‘Pera’/‘Rangpur’ lime | | | | |
| 1 | 25.2 | 4.8 | 29.2 | 3.6 |
| 2 | 29.1 | 3.7 | 24.3 | 5.1 |
| 3 | 25.4 | 4.8 | 26.4 | 4.5 |
| 4 | 28.5 | 3.8 | 30.1 | 3.4 |
| 5 | 28.5 | 3.8 | 29.2 | 3.6 |
| 6 | 25.3 | 4.8 | 26.7 | 4.4 |
| 7 | 25.2 | 4.8 | 30.1 | 3.4 |
| 8 | 28.2 | 3.9 | 26.9 | 4.3 |
| 9 | 21.5 | 5.9 | 28.9 | 3.7 |
| 10 | 23.2 | 5.4 | 27.3 | 4.2 |
| 11 | 25.2 | 4.8 | 28.4 | 3.9 |
| 12 | 23.2 | 5.4 | 28.6 | 3.8 |
| 13 | 20.1 | 6.4 | 28.4 | 3.9 |
| 14 | 25.9 | 4.6 | 28.9 | 3.7 |
| 15 | 28.3 | 3.9 | 24.6 | 5.0 |
| *Citrus* ×*sinensis* ‘Tobias’/‘Rangpur’ lime | | | | |
| 1 | 20.5 | 6.2 | 27.8 | 4.1 |
| 2 | 21.6 | 5.9 | 28.9 | 3.7 |
| 3 | 22.1 | 5.7 | 25.4 | 4.8 |
| 4 | 33.4 | 2.4 | 29.6 | 3.5 |
| 5 | 24.6 | 5.0 | 30.2 | 3.3 |
| 6 | 21.2 | 6.0 | 30.1 | 3.4 |
| 7 | 27.9 | 4.0 | 29.8 | 3.5 |
| 8 | 22.9 | 5.5 | 30.1 | 3.4 |
| 9 | 20.3 | 6.3 | 29.9 | 3.4 |
| *Microcitrus australasica* ‘True Sanguinea’*‘/‘*Rangpur’ lime | | | | |
| 1 | nd^c^ | nd | 31.2 | 3.0 |
| 2 | nd | nd | 32.3 | 2.7 |
| 3 | 33.4 | 2.4 | 32.3 | 2.7 |
| 4 | nd | nd | 33.2 | 2.4 |
| 5 | nd | nd | 32.1 | 2.8 |
| 6 | 33.2 | 2.4 | 30.0 | 3.4 |
| 7 | nd | nd | 31.9 | 2.8 |
| 8 | D^d^ | D | D | D |
| 9 | nd | nd | 33.9 | 2.2 |
| 10 | 32.5 | 2.6 | 30.4 | 3.3 |
| 11 | nd | nd | 32.6 | 2.6 |
| ‘Faustrimedin’ hybrid; *C*. x *oliveri* /‘Rangpur’ lime | | | | |
| 1 | 29.9 | 3.4 | 31.8 | 2.9 |
| 2 | nd | nd | 31.2 | 3.0 |
| 3 | 33.9 | 2.2 | 31.6 | 2.9 |
| 4 | nd | nd | 31.9 | 2.8 |
| 5 | nd | nd | 27.6 | 4.1 |
| 6 | nd | nd | 31.0 | 3.1 |
| 7 | 31.3 | 3.0 | 28.2 | 3.9 |
| 8 | nd | nd | 33.9 | 2.2 |
| 9 | nd | nd | 33.8 | 2.6 |
| 10 | 25.9 | 4.6 | 33.3 | 2.4 |
| *Microcitrus inodora/‘*Rangpur’ lime | | | | |
| 1 | D | D | D | D |
| 2 | nd | nd | 30.0 | 3.4 |
| 3 | D | D | D | D |
| 4 | nd | nd | 33.2 | 2.4 |
| 5 | nd | nd | 33.5 | 2.3 |
| 6 | D | D | D | D |
| 7 | 21.9 | 5.8 | 26.1 | 4.6 |
| 8 | nd | nd | 26.2 | 4.5 |
| 9 | nd | nd | 33.5 | 2.3 |
| 10 | nd | nd | 28.7 | 3.8 |
| 11 | nd | nd | 26.4 | 4.5 |
| 12 | 21.5 | 5.9 | 30.1 | 3.4 |
| 13 | 32.5 | 2.6 | 26.1 | 4.6 |
| 14 | D | D | D | D |
| *Microcitrus virgata* hybrid/‘Rangpur’ lime | | | | |
| 1 | nd | nd | 29.3 | 3.6 |
| 2 | d | d | d | d |
| 3 | d | d | d | d |
| 4 | d | d | d | d |
| 5 | d | d | d | d |
| 6 | nd | nd | 30.2 | 3.3 |
| 7 | D | D | D | D |
| 8 | nd | nd | 27.9 | 4.0 |
| 9 | nd | nd | 29.8 | 3.5 |
| 10 | nd | nd | 27.6 | 4.1 |
| *Microcitrus warburgiana/‘*Rangpur’ lime | | | | |
| 1 | nd | nd | 30.1 | 3.4 |
| 2 | nd | nd | 30.2 | 3.3 |
| 3 | nd | nd | 29.9 | 3.4 |
| 4 | D | D | D | D |
| 5 | nd | nd | 30.1 | 3.4 |
| 6 | D | D | D | D |
| 7 | nd | nd | 30.2 | 3.3 |
| 8 | nd | nd | 30.3 | 3.3 |
| 9 | D | D | D | D |
| *Microcitrus papuana/‘*Rangpur’ lime | | | | |
| 1 | D | D | D | D |
| 2 | nd | nd | 30.2 | 3.3 |
| 3 | nd | nd | 32.3 | 2.7 |
| 4 | nd | nd | 33.8 | 2.3 |
| 5 | D | D | D | D |
| 6 | D | D | D | D |
| 7 | nd | nd | 28.6 | 3.8 |
| 8 | D | D | D | D |
| *Microcitrus australis/‘*Rangpur’ lime | | | | |
| 1 | D | D | D | D |
| 2 | nd | nd | 30.1 | 3.4 |
| 3 | nd | nd | 30.7 | 3.2 |
| 4 | nd | nd | 30.3 | 3.3 |
| 5 | nd | nd | 30.2 | 3.3 |
| 6 | nd | nd | 29.9 | 3.4 |
| 7 | nd | nd | 31.0 | 3.1 |
| 8 | D | D | D | D |
| 9 | nd | nd | 29.9 | 3.4 |
| 10 | nd | nd | 30.1 | 3.4 |
| *Microcitrus* sp. x *Eremocitrus glauca* hybrid/‘Rangpur’ lime | | | | |
| 1 | nd | nd | 29.0 | 3.7 |
| 2 | nd | nd | 28.9 | 3.7 |
| 3 | nd | nd | 34.0 | 2.2 |
| 4 | nd | nd | 31.7 | 2.9 |
| 5 | nd | nd | 29.1 | 3.7 |
| 6 | nd | nd | 34.0 | 2.2 |
| 7 | nd | nd | 30.3 | 3.3 |
| *Eremocitrus glauca* x *Citrus* ×*sinensis* hybrid*/‘*Rangpur’ lime | | | | |
| 1 | nd | nd | 29.0 | 3.7 |
| 2 | nd | nd | 27.2 | 4.2 |
| 3 | nd | nd | 28.1 | 4.0 |
| 4 | nd | nd | 26.8 | 4.3 |
| 5 | nd | nd | 26.6 | 4.4 |
| 6 | nd | nd | 28.9 | 3.7 |
| 7 | nd | nd | 30.6 | 3.2 |
| 8 | D | D | D | D |
| 9 | nd | nd | 26.7 | 4.4 |
| 10 | nd | nd | 30.4 | 3.3 |
| 11 | nd | nd | 29.7 | 3.5 |
| 12 | nd | nd | 29.9 | 3.4 |
| *Eremocitrus glauca* x *Microcitrus* sp. hybrid/‘Rangpur’ lime | | | | |
| 1 | nd | nd | 33.4 | 2.4 |
| 2 | nd | nd | 33.3 | 2.4 |
| 3 | nd | nd | 33.4 | 2.4 |
| 4 | nd | nd | 33.0 | 2.5 |
| 5 | nd | nd | 33.0 | 2.5 |
| 6 | nd | nd | 33.7 | 2.3 |
| 7 | nd | nd | 33.5 | 2.3 |
| 8 | nd | nd | 33.9 | 2.2 |

^a^Ct: Cycle threshold.

^b^Log: Las titer in log10 of amplicon copies per gram of plant tissue estimated based on a standard curve as described by Lopes et al., 2013.

^c^nd: Non-detected.

^d^D: Plant died.

**Supplementary Table 11.** ‘*Candidatus* Liberibacter asiaticus’ detection in scions from each plant of Category 3 accessions plus sweet orange controls. Bark samples were taken at different distances from the rootstock (5 cm, 30 cm and at the canopy) at 24 months after the inoculation (24 MAI).

| **Plant number** | **Scion** | | | | | |
| --- | --- | --- | --- | --- | --- | --- |
|  | **Bark** | | | | | |
|  | **5 cm** | | **30 cm** | | **Canopy (21-152 cm)** | |
|  | **Ct^a^** | **Log^b^** | **Ct** | **Log** | **Ct** | **Log** |
| *Citrus* × s*inensis* ‘Pera’ / Rangpur lime | | | | | | |
| 1 | 21.5 | 5.9 | 25.6 | 4.7 | 23.1 | 5.5 |
| 2 | 24.1 | 5.2 | 29.6 | 3.5 | 25.6 | 4.7 |
| 3 | 23.5 | 5.3 | 23.1 | 5.5 | 22.8 | 5.6 |
| 4 | 25.4 | 4.8 | 25.6 | 4.7 | 24.6 | 5.0 |
| 5 | 23.9 | 5.2 | 28.0 | 4.0 | 24.3 | 5.1 |
| 6 | 25.4 | 4.8 | 26.4 | 4.5 | 25.8 | 4.7 |
| 7 | 26.9 | 4.3 | 26.5 | 4.4 | 28.0 | 4.0 |
| 8 | 28.1 | 4.0 | 28.1 | 4.0 | 21.5 | 5.9 |
| 9 | 26.1 | 4.6 | 26.5 | 4.4 | 22.9 | 5.5 |
| 10 | 29.1 | 3.7 | 28.1 | 4.0 | 25.4 | 4.8 |
| 11 | 23.6 | 5.3 | 27.1 | 4.3 | 25.8 | 4.7 |
| 12 | 21.5 | 5.9 | 27.1 | 4.3 | 23.6 | 5.3 |
| 13 | 28.1 | 4.0 | 22.5 | 5.6 | 21.5 | 5.9 |
| 14 | 26.9 | 4.3 | 23.4 | 5.4 | 22.5 | 5.6 |
| 15 | 25.1 | 4.9 | 21.1 | 6.1 | 28.0 | 4.0 |
| 16 | 23.6 | 5.3 | 23.5 | 5.3 | 26.4 | 4.5 |
| 17 | 26.5 | 4.4 | 26.8 | 4.4 | 25.8 | 4.7 |
| 18 | 27.2 | 4.2 | 25.4 | 4.8 | 27.1 | 4.3 |
| 19 | 25.3 | 4.8 | 29.2 | 3.6 | 29.4 | 3.6 |
| 20 | 24.1 | 5.2 | 28.4 | 3.9 | 27.5 | 4.1 |
| 21 | 25.2 | 4.8 | 25.6 | 4.7 | 29.2 | 3.6 |
| 22 | 23.4 | 5.4 | 21.3 | 6.0 | 28.4 | 3.9 |
| 23 | 25.6 | 4.7 | 25.3 | 4.8 | 24.5 | 5.0 |
| 24 | 21.2 | 6.0 | 26.5 | 4.4 | 26.5 | 4.4 |
| 25 | 23.1 | 5.5 | 25.1 | 4.9 | 25.4 | 3.3 |
| 26 | 25.4 | 4.8 | 25.1 | 4.9 | 25.3 | 4.8 |
| 27 | 26.5 | 4.4 | 28.9 | 3.7 | 21.5 | 5.9 |
| 28 | 25.4 | 4.8 | 24.7 | 5.0 | 27.9 | 4.0 |
| 29 | 23.2 | 5.4 | 25.6 | 4.7 | 26.5 | 4.4 |
| 30 | 23.3 | 5.4 | 23.5 | 5.3 | 24.5 | 5.0 |
| 31 | 21.5 | 5.9 | 21.3 | 6.0 | 23.2 | 5.4 |
| 32 | 25.9 | 4.6 | 25.6 | 4.7 | 25.1 | 4.9 |
| 33 | 28.2 | 3.9 | 21.6 | 5.9 | 25.9 | 4.6 |
| 34 | 29.3 | 3.6 | 25.6 | 4.7 | 25.2 | 4.8 |
| 35 | 27.8 | 4.1 | 25.2 | 4.8 | 23.6 | 5.3 |
| 36 | 28.9 | 3.7 | 23.5 | 5.3 | 23.1 | 5.5 |
| 37 | 24.5 | 5.0 | 25.6 | 4.7 | 22.1 | 5.8 |
| 38 | 26.7 | 4.4 | 24.8 | 5.0 | 22.5 | 5.6 |
| 39 | 28.8 | 3.8 | 25.6 | 4.7 | 25.9 | 4.6 |
| 40 | 29.1 | 3.7 | 24.1 | 5.2 | 25.4 | 4.8 |
| 41 | 25.4 | 4.8 | 25.4 | 4.8 | 23.2 | 5.4 |
| *Citrus* ×*sinensis* ‘Tobias’/ Rangpur lime | | | | | | |
| 1 | 29.8 | 3.5 | 23.3 | 5.4 | 23.1 | 5.5 |
| 2 | 27.1 | 4.3 | 21.3 | 6.0 | 25.5 | 4.7 |
| 3 | 26.4 | 4.5 | 29.9 | 3.4 | 22.9 | 5.5 |
| 4 | 25.1 | 4.9 | 24.6 | 5.0 | 24.6 | 5.0 |
| 5 | 29.3 | 3.6 | 28.1 | 4.0 | 21.8 | 5.9 |
| 6 | 29.3 | 3.6 | 23.5 | 5.3 | 27.1 | 4.3 |
| 7 | 30.1 | 3.4 | 24.1 | 5.2 | 21.6 | 5.9 |
| 8 | 21.2 | 6.0 | 28.1 | 4.0 | 22.3 | 5.7 |
| 9 | 22.3 | 5.7 | 24.1 | 5.2 | 25.1 | 4.9 |
| *Microcitrus warbugiana /* Rangpur lime | | | | | | |
| 1 | 30.0 | 3.4 | nd^c^ | nd | nd | nd |
| 2 | 31.8 | 2.9 | nd | nd | nd | nd |
| 3 | 32.1 | 2.8 | nd | nd | nd | nd |
| 4 | D^d^ | D | D | D | D | D |
| 5 | nd | nd | nd | nd | nd | nd |
| 6 | D | D | D | D | D | D |
| 7 | 32.9 | 2.5 | nd | nd | nd | nd |
| 8 | nd | nd | nd | nd | nd | nd |
| 9 | D | D | D | D | D | D |
| *Microcitrus papuana /* Rangpur lime | | | | | | |
| 1 | D | D | D | D | D | D |
| 2 | 32.9 | 2.5 | nd | nd | nd | nd |
| 3 | nd | nd | nd | nd | nd | nd |
| 4 | nd | nd | nd | nd | nd | nd |
| 5 | D | D | D | D | D | D |
| 6 | D | D | D | D | D | D |
| 7 | 32.1 | 2.8 | nd | nd | nd | nd |
| 8 | D | D | D | D | D | D |
| *Microcitrus australis /* Rangpur lime | | | | | | |
| 1 | D | D | D | D | D | D |
| 2 | 32.3 | 2.7 | nd | nd | nd | nd |
| 3 | 35.3 | 1.8 | nd | nd | nd | nd |
| 4 | 32.1 | 2.8 | nd | nd | nd | nd |
| 5 | 34.8 | 2.0 | nd | nd | nd | nd |
| 6 | 32.6 | 2.6 | nd | nd | nd | nd |
| 7 | nd | nd | nd | nd | nd | nd |
| 8 | D | D | D | D | D | D |
| 9 | 31.2 | 3.0 | nd | nd | nd | nd |
| 10 | 31.0 | 3.1 | nd | nd | nd | nd |
| *Microcitrus* x *Eremocitrus* hybrid / Rangpur lime | | | | | | |
| 1 | 29.9 | 3.4 | 28.1 | 4.0 | nd | nd |
| 2 | 29.9 | 3.4 | 25.9 | 4.6 | nd | nd |
| 3 | 30.1 | 3.4 | nd | nd | nd | nd |
| 4 | 30.8 | 3.2 | 29.4 | 3.6 | nd | nd |
| 5 | 33.1 | 2.5 | nd | nd | nd | nd |
| 6 | nd | nd | nd | nd | nd | nd |
| 7 | 30.8 | 3.2 | nd | nd | nd | nd |
| *Eremocitrus glauca /* Rangpur lime | | | | | | |
| 1 | 30.1 | 3.4 | nd | nd | nd | nd |
| 2 | 32.1 | 2.8 | nd | nd | nd | nd |
| 3 | nd | nd | nd | nd | nd | nd |
| 4 | 30.1 | 3.4 | nd | nd | nd | nd |
| 5 | 29.8 | 3.5 | nd | nd | nd | nd |
| 6 | 35.2 | 1.8 | nd | nd | nd | nd |
| 7 | 32.1 | 2.8 | nd | nd | nd | nd |
| *E. glauca* x *C.* ×*sinensis* hybrid */* Rangpur lime | | | | | | |
| 1 | 31.1 | 3.1 | 30.5 | 3.2 | nd | nd |
| 2 | 31.2 | 3.0 | 29.4 | 3.6 | nd | nd |
| 3 | 33.7 | 2.3 | nd | nd | nd | nd |
| 4 | 30.9 | 3.1 | nd | nd | nd | nd |
| 5 | 29.1 | 3.7 | 30.9 | 3.1 | nd | nd |
| 6 | 30.2 | 3.3 | 31.5 | 2.9 | nd | nd |
| 7 | 30.9 | 3.1 | nd | nd | nd | nd |
| 8 | D | D | D | D | D | D |
| 9 | 31.5 | 2.9 | nd | nd | nd | nd |
| 10 | 32.1 | 2.8 | nd | nd | nd | nd |
| 11 | 32.1 | 2.8 | nd | nd | nd | nd |
| 12 | 30.7 | 3.2 | nd | nd | nd | nd |
| *Eremocitrus* x *Microcitrus* hybrid / Rangpur lime | | | | | | |
| 1 | 32.5 | 2.6 | nd | nd | nd | nd |
| 2 | 33.0 | 2.5 | nd | nd | nd | nd |
| 3 | 31.0 | 3.1 | nd | nd | nd | nd |
| 4 | 32.3 | 2.7 | nd | nd | nd | nd |
| 5 | 31.2 | 3.0 | nd | nd | nd | nd |
| 6 | 33.2 | 2.4 | nd | nd | nd | nd |
| 7 | 31.5 | 2.9 | nd | nd | nd | nd |
| 8 | 31.9 | 2.8 | nd | nd | nd | nd |

^a^Ct: Cycle threshold.

^b^Log: Las titer in log10 of amplicon copies per gram of plant tissue estimated based on a standard curve as described by Lopes et al., 2013.

^c^nd: Non-detected.

^d^D: Plant died.
